# The transcriptome landscape of 3D-cultured placental trophoblasts reveals activation of TLR2 and TLR3/7 in response to low *Trypanosoma cruzi* parasite exposure

**DOI:** 10.3389/fmicb.2023.1256385

**Published:** 2023-09-20

**Authors:** Erica Silberstein, Charles C. Chung, Alain Debrabant

**Affiliations:** ^1^Laboratory of Emerging Pathogens, Office of Blood Research and Review, Center for Biologics Evaluation and Research, Food and Drug Administration, Silver Spring, MD, United States; ^2^High-performance Integrated Virtual Environment Team, Office of Biostatistics and Epidemiology, Center for Biologics Evaluation and Research, Food and Drug Administration, Silver Spring, MD, United States

**Keywords:** congenital Chagas disease, *Trypanosoma cruzi*, 3D-trophoblasts, resistance to infection, transcriptome analysis, Toll-like receptors

## Abstract

Vertical transmission of *Trypanosoma cruzi* (*T. cruzi*) become a globalized health problem accounting for 22% of new cases of Chagas disease (CD). Congenital infection is now considered the main route of CD spread in non-endemic countries where no routine disease testing of pregnant women is implemented. The main mechanisms that lead to fetal infection by *T. cruzi* remain poorly understood. Mother-to-child transmission may occur when bloodstream trypomastigotes interact with the syncytiotrophoblasts (SYNs) that cover the placenta chorionic villi. These highly specialized cells function as a physical barrier and modulate immune responses against pathogen infections. To model the human placenta environment, we have previously used a three-dimensional (3D) cell culture system of SYNs that exhibits differentiation characteristics comparable to placental trophoblasts. Further, we have shown that 3D-grown SYNs are highly resistant to *T. cruzi* infection. In this work, we used RNA sequencing and whole transcriptome analysis to explore the immunological signatures that drive SYNs’ infection control. We found that the largest category of differentially expressed genes (DEGs) are associated with inflammation and innate immunity functions. Quantitative RT-PCR evaluation of selected DEGs, together with detection of cytokines and chemokines in SYNs culture supernatants, confirmed the transcriptome data. Several genes implicated in the Toll-like receptors signaling pathways were upregulated in 3D-grown SYNs. In fact, TLR2 blockade and TLR3/7 knockdown stimulated *T. cruzi* growth, suggesting that these molecules play a significant role in the host cell response to infection. Ingenuity Pathway Analysis of DEGs predicted the activation of canonical pathways such as S100 protein family, pathogen induced cytokine storm, wound healing, HIF1α signaling and phagosome formation after *T. cruzi* exposure. Our findings indicate that SYNs resist infection by eliciting a constitutive pro-inflammatory response and modulating multiple defense mechanisms that interfere with the parasite’s intracellular life cycle, contributing to parasite killing and infection control.

## 1. Introduction

Chagas disease (CD) is a lifelong zoonotic disease caused by the protozoan parasite *Trypanosoma cruzi (T. cruzi)* that affects six to seven million people worldwide (Perez-Molina and Molina, 2018). CD is endemic in Latin America where the parasite is mainly transmitted to humans and domestic animals by infected triatomine bugs. *T. cruzi* infection can also be transmitted through blood transfusion, organ transplantation, and from mother-to-baby. In non-endemic areas, *T. cruzi* congenital infection has become the main active route of CD spread (Carlier et al., 2019; Rios et al., 2020) with cases reported in the US, Canada, Europe and Japan (Imai et al., 2019; Irish et al., 2022; Navarro et al., 2022).

An estimated 43,000 *T. cruzi*-infected women of reproductive age live the United States where the vertical transmission rate is approximately 1–5% (Edwards et al., 2019; Irish et al., 2022). Congenital infections can occur both during the acute and chronic phases of maternal infection, but transmission rates increase in pregnant women with high parasitemia (Carlier et al., 2020; Klein et al., 2021). Most infected newborns do not present clinical signs at birth although in exceptional cases, they may experience severe symptoms that can lead to death (Matthews et al., 2022). Early diagnosis and therapeutic interventions are crucial to prevent cardiac or gastrointestinal complications later in life (Carlier et al., 2019; Perez-Zetune et al., 2020).

During pregnancy, the placenta forms a physical barrier between the mother and developing fetus. A single layer of multinucleated cells, the syncytiotrophoblasts (SYNs), covers the placenta free-floating chorionic villous trees that are in direct contact with maternal blood. The SYNs not only allow the exchange of nutrients and waste but also produce pregnancy hormones and immunoregulatory molecules that prevent pathogen fetal infections (Arora et al., 2017; Ander et al., 2019; Kemmerling et al., 2019).

Previous studies, based on histological analysis of infected human placentas and placental explants, indicate that *T. cruzi* induces destruction and detachment of SYNs (Duaso et al., 2010; Keogh et al., 2021; Mezzano et al., 2022). Other investigators have found parasitic lesions in placental tissues at the marginal zone (Fernandez-Aguilar et al., 2005). These observations suggest that *T. cruzi* may reach umbilical and fetal capillaries either by infecting SYNs or through invasion of the marginal sinus epithelial cells (Carlier et al., 2020; Avalos-Borges et al., 2022).

The main mechanisms and immunoregulatory factors that modulate *T. cruzi* fetal infection have yet to be determined. Several animal and *ex vivo* models have been used to study CD congenital transmission (Avalos-Borges et al., 2022). For instance, modulation of the NF-κB and Toll-like receptors (TLRs) pathways, as well as the role of the placental barrier integrity in parasite infection have been evaluated in chorionic villi explants (Diaz-Lujan et al., 2016; Liempi et al., 2016; Castillo et al., 2017a,b) and cord blood cells (Ait Djebbara et al., 2023). Yet, the ability to reproduce parasite infection in an environment that resembles the complex architecture of the human placenta continues to be a challenge for the field (Barrila et al., 2018; Torres-Vargas et al., 2018; Ander et al., 2019).

To simulate the human placenta environment, we and others have used the rotating wall vessel bioreactor (RWV) platform to culture SYNs (McConkey et al., 2016; Corry et al., 2017; Silberstein et al., 2021). When grown under the dynamic culture conditions provided by this system, SYNs differentiate into 3D tissue-like spheroids that form syncytia, produce placental-specific hormones and show a transcriptome profile strikingly similar to primary human SYNs (McConkey et al., 2016). Furthermore, and consistent with the intrinsic protective function of the placenta (Hoo et al., 2020), we have shown that 3D-grown SYNs are highly resistant to *T. cruzi* infection (Silberstein et al., 2021).

In this study, we sought to explore *T. cruzi*-host cell interactions and determine the immunological hallmark of 3D-grown SYNs in response to parasite infection using RNA sequencing followed by whole transcriptome analysis and functional studies. Our data suggest that SYNs’ exposure to *T. cruzi* results in the activation of multiple defense mechanisms related to parasite sensing, internalization and phagocytosis, which can ultimately culminate with killing of the parasite by the host cell.

## 2. Materials and methods

### 2.1. Cells

2D-cultured JEG-3 cells (2D SYNs; ATCC® HTB-36™, American Type Culture Collection, VA), human brain microvascular endothelial cells [HBMECs; (Coyne et al., 2007)], LLC-MK2 cells (ATCC® CCL-7™; American Type Culture Collection, VA), and three-dimensional (3D) cultures of JEG-3 cells (3D SYNs) were grown as described previously (Silberstein et al., 2021). Experiments with 3D SYNs and 3D HBMECs were carried out between day 20–22 and day 4–6 after culture initiation, respectively. The number of viable cells was determined using the CellTiter-Fluor™ (for 2D SYNs) or the CellTiter-Glo™ 3D (for 3D SYNs) cell viability assays following manufacturer’s instructions (Promega, WI).

### 2.2. *Trypanosoma cruzi* propagation and infection

Trypomastigotes of the *T. cruzi* strain Colombiana expressing nanoluciferase [TcCOL-NLuc (Silberstein et al., 2018)] were harvested from culture supernatants of infected LLC-MK2 cells and the number of parasites was determined using a Cellometer K2 Fluorescent Viability Cell Counter, following manufacturer’s instructions (Nexcelom Bioscience, MA). Infection of 2D SYNs was performed in 96-well plates (seeded at 1×10^4^ cells /well) or T75 culture flasks using a ratio of 5 parasites/cell (MOI = 5). For cultures grown in 3D, spheroids were plated in 96-well plates (100 μL/well) or 6 well plates (2 mL/well) and cells were infected at MOI = 5. *T. cruzi* growth was monitored in 96-well plates using the Nano-Glo® Luciferase Assay following manufacturer’s instructions (Promega, WI) as previously described (Silberstein et al., 2021).

### 2.3. RNA sequencing and data processing

Total RNA was extracted from harvested 3D and 2D-cultured SYNs, and 3D-cultured HBMECs 18 h post-infection, using the PureLink™ RNA mini kit (Invitrogen, CA). RNA integrity number (RIN) and concentration for each sample was determined with the Agilent 2,100 Bioanalyzer (Agilent Technologies, CA). All RIN values were within the acceptable range (RIN ≥ 9). Two to four replicates from cells exposed and not exposed to *T. cruzi* were used to perform transcriptome sequencing at Novogene^1^ according to their protocols. Briefly, oligo d(T) beads were employed to perform mRNA enrichment, which were then fragmented randomly and converted into cDNA. Poly(A) tails were added and enriched by PCR to generate a cDNA library using NEBNext® Ultra™ RNA Library Prep Kit for Illumina® (NEB, MA). Illumina HiSeq 2,500 was used to perform paired-end sequencing of 150 bp reads. The read quality was analyzed with fastp (Chen et al., 2018) trimming polyG tail, polyX in 3′ ends and filtered reads shorter than length 20. Quality passed reads were aligned to the human reference genome (GRCh38.p13) using HISAT2 v2.1.0 (Kim et al., 2019). The mapped reads were counted with featureCounts (Liao et al., 2014) for genes annotated in the GCF_000001405.39 annotation file from NCBI.

### 2.4. Differential gene expression and pathway analysis

The differential gene expression (DGE) analysis was conducted for the 3D SYNs (*n* = 4) and 2D SYNs (*n* = 2), and for the 3D HBMECs (*n* = 3) and 2D SYNs (*n* = 2), unexposed or exposed to the parasite for 18 h using the DESeq2 R package (Love et al., 2014). The alpha and the fold change thresholds were set to a Benjamin–Hochberg-adjusted *p*-value <0.05 and 1.5, respectively for significance in the DGE test. Read counts were normalized using the median of ratios method. DEG cluster analysis was performed using the pheatmap R package.

The log_2_ (fold-change) expression values of DEGs and *p*-adjusted values were uploaded into the Ingenuity Pathway Analysis software [IPA; QIAGEN (Kramer et al., 2014)] to determine activated or inhibited canonical pathways. *Z*-scores were used to predict pathway activation (*z*-score ≥ 2) or inhibition (*z*-score ≤ 2). Statistical significance was calculated using a right-tailed Fisher Exact test and *p-*-values were adjusted for multiple hypothesis testing using the Benjamin–Hochberg method. An absolute B-H *p*-value <0.05 was considered significant.

### 2.5. Quantitative RT-PCR

Following purification, total RNA was reverse- transcribed into cDNA using the iScript gDNA clear cDNA synthesis kit (Bio-Rad Laboratories, CA) and 0.5 to 1 μg of RNA template. Quantitative PCR was carried out using the SsoAdvanced™-Universal SYBR® Green Supermix (Bio-Rad Laboratories, CA) in a CFX96 touch real-time PCR detection system (Bio-Rad Laboratories, CA). Eighty-four genes associated with host immune defenses were profiled using the Innate and Adaptative Immune Responses PrimePCR Array (Bio-Rad Laboratories, CA). The cycling protocol included an initial activation step at 95°C for 2 min, followed by 40 cycles of 5 s at 95°C and 30 s at 60°C. Finally, a melt curve analysis was run for 5 s/step between 65 and 95°C with 0.5°C increments. Data were analyzed with CFX Manager Software. Gene expression was determined by the ∆C_t_ method with samples normalized to human GAPDH (Livak and Schmittgen, 2001).

### 2.6. Bio-Plex assay for cytokines profiling

Cytokine levels in culture supernatants of 3D SYNs (unexposed or exposed to *T. cruzi*) were measured using a Bio-Plex Pro Human Cytokine 17-plex Assay (Bio-Rad Laboratories, CA), according to the manufacturer’s instructions. Briefly, 50 μL of coupled magnetic beads mixture was added to each well. After three washes, 50 μL of undiluted culture supernatants and serial dilutions of standards were added to duplicate wells. The plate was incubated on a microplate orbital shaker at 850 rpm for 2 h and next washed three times before adding 50 μL of detection antibodies conjugated with biotin. Following a 30 min incubation, wells were washed again three times, and 50 μL streptavidin-phycoerythin solution was added. After washing three times, beads were resuspended in assay buffer and incubated for 10 min. All incubations were carried out at room temperature. Data were acquired using a Luminex 200 instrument (Luminex Corporation, TX) and analyzed with the BioPlex Pro software (Bio-Rad Laboratories, CA). Concentration of each analyte in pg/ml was calculated from corresponding standard curves.

### 2.7. TLR2 blockade

2D SYNs (7.5×10^3^ cells) were seeded into each well of 96 well plates. After 1 h incubation with 5 mg/mL anti-hTLR2 (InvivoGen, CA) or RecombiMAb human IgG1 (LALA-PG) isotype control (Bio X Cell, NH), cells were infected with TcCOL-NLuc trypomastigotes using a ratio of 5 parasites/cell. After an overnight incubation, free parasites were removed, and cells were washed three times with culture media containing 2% fetal bovine serum (FBS). Next, 100 μL of fresh culture media containing 5 mg/mL of antibodies were added per well and parasite growth was determined at 48- and 72 h post-infection by measuring nanoluciferase (NLuc) activity in triplicate wells as described (Silberstein et al., 2021). Cells from four replicate wells were pooled and total RNA was extracted at identical time points. Quantitative RT-PCR to measure cytokines mRNA was carried out using the SsoAdvanced™-Universal SYBR® Green Supermix (Bio-Rad Laboratories, CA) and primer sets specific to human IL6 and TNF-α (Bio-Rad Laboratories, CA). Gene expression was determined by the ∆C_t_ method with samples normalized to human GAPDH (Livak and Schmittgen, 2001).

### 2.8. siRNA mediated knockdown of TLR3 and TLR7

On-target plus non-targeting siRNA control pool and on-target plus human TLR3 and TLR7 siRNA smartpools were purchased from Horizon (Lafayette, CO). Transfections were performed according to the manufacturer’s instructions. In brief, 2D SYNS (1×10^4^) were seeded into each well of 96-well plate using antibiotic-free complete medium. Each transfection was carried out in three replicate wells using a 100 nM final concentration of siRNA (50 nM of TLR3 siRNA and 50 nM of TLR7 siRNA) mixed with 0.3 μL/well of DharmaFECT 3 transfection reagent (Horizon, CO). After 24 h, cells were infected with TcCOL-NLuc trypomastigotes using a ratio of 5 parasites/cell. Free parasites were removed after 24 h post-infection and cells were washed three times with culture media. Parasite growth was determined at 48- and 72 h post-infection by measuring nanoluciferase (NLuc) activity as described (Silberstein et al., 2021). Cells from triplicate wells were pooled and total RNA was extracted at identical time points. Quantitative RT-PCR to verify TLRs silencing and to measure cytokines mRNA, was carried out using the SsoAdvanced™-Universal SYBR® Green Supermix (Bio-Rad Laboratories, CA) and primer sets specific to human TLR3, TLR7, IRF3, IRF7, IFN-α1 and IFN-β1 (Bio-Rad Laboratories, CA). Gene expression was determined by the ∆C_t_ method with samples normalized to human GAPDH (Livak and Schmittgen, 2001).

### 2.9. Statistical analysis

All bar graphs are displayed as means ± SD. Statistical analysis of differences between mean values of groups was determined by the unpaired two-tailed Student’s *t*-test, using GraphPad Prism software version 9.0.0. A *p* value <0.05 was considered significant.

## 3. Results

### 3.1. Transcriptomic profiling reveals distinct gene expression changes in 3D-grown SYNs compared to 2D-grown

**Figure 1:**
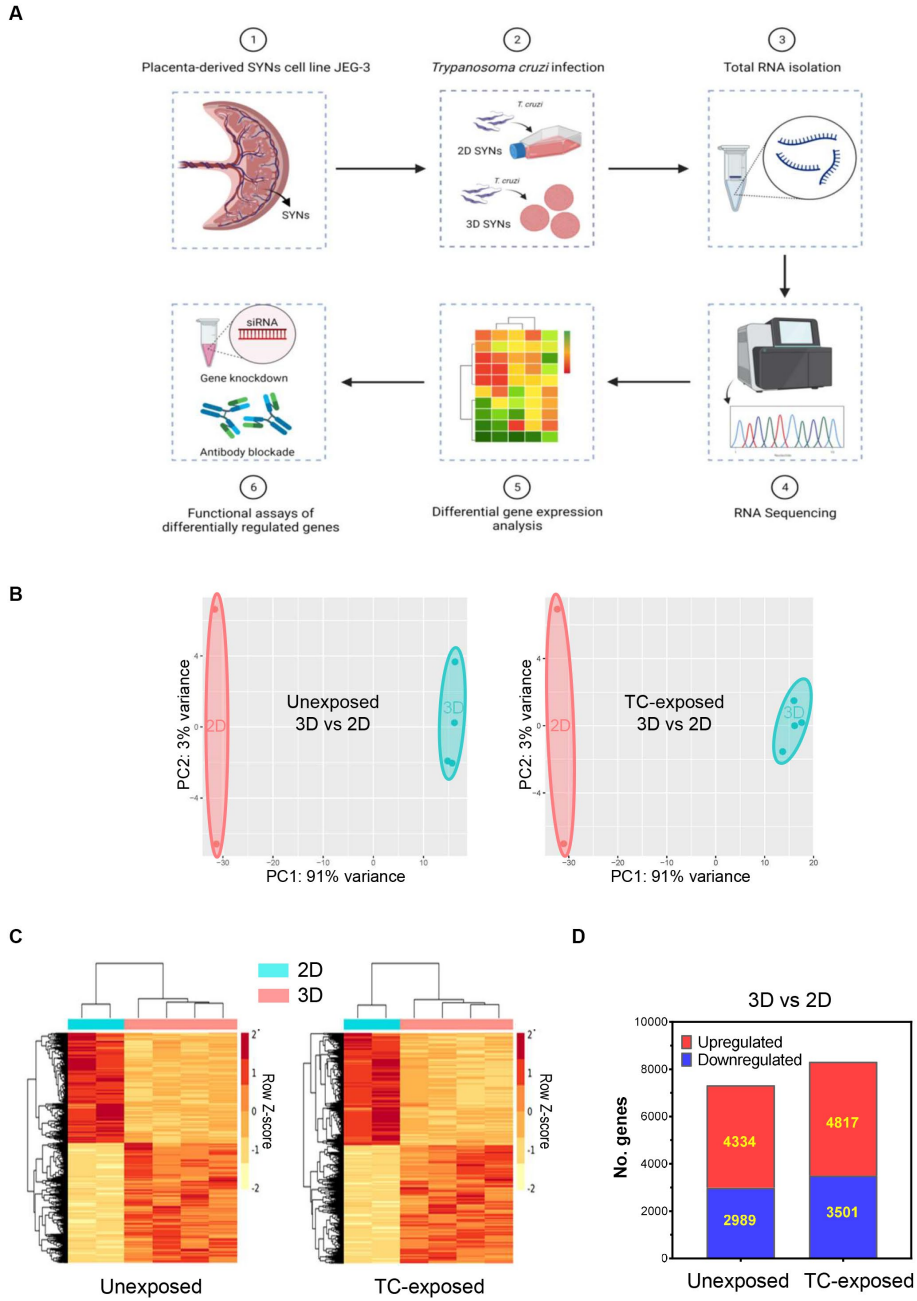
Alterations in global gene expression profiles of 3D and 2D-grown SYNs. 2D or 3D SYNs were infected with *T. cruzi* at MOI = 5 and harvested at 18 hpi for transcriptomic analysis by RNA-Seq. Differentially expressed genes were identified using the DEseq2 software. The top signaling pathways activated in 3D SYNs were predicted through Ingenuity Pathway Analysis. Functional studies were carried out to verify the impact of selected genes in the regulation of the identified biological pathways. **(A)** Experimental workflow for the whole transcriptome analysis of 2D and 3D-grown SYNs cultures unexposed or exposed to *T. cruzi*. **(B)** Principal component analysis (PCA) of RNA-Seq data of 2D and 3D-cultured SYNs unexposed or exposed to *T. cruzi*. **(C)** Hierarchical cluster analysis of normalized read counts of differentially expressed genes between 3D and 2D SYNs unexposed or exposed to *T. cruzi*. **(D)** Number of significantly up- and down-regulated genes in 3D SYNs with respect to 2D SYNs (fold-change ≥1.5 and FDR < 0.05).

To identify the possible mechanisms responsible for the resistance of 3D-grown SYNs to *T. cruzi* infection, we performed high throughput RNA sequencing (RNA-Seq) on four independent SYNs cultures grown in 3D and two independent 2D SYNs cultures, unexposed or exposed to the parasite for 18 h (Figure 1A). We used a ratio of 5 parasites/cell, conditions representative of a chronic infection characterized by moderate to low parasitemia (Carlier et al., 2020).

When we compared the transcriptome profile of *T. cruzi* -exposed 2D SYNs with unexposed 2D cultures, and *T. cruzi* -exposed 3D SYNs with unexposed 3D cultures, we observed few changes in the abundance of transcripts. These results correlate with previous findings that showed a weak modulation of host cell transcription in response to trypomastigotes invasion (Vaena de Avalos et al., 2002; Perumal et al., 2023).

With the goal of finding the main factors that drive 3D SYNs resistance to the parasite, we next studied the transcriptome profile of 3D SYNs compared to 2D SYNs, in both unexposed and *T. cruzi* exposed cultures. Interestingly, we found many transcriptional changes in 3D SYNs compared to 2D SYNs. As illustrated by the principal component analysis (PCA), two groups could be clearly discriminated with samples closely clustering together in each group (Figure 1B, 3D vs. 2D, unexposed or *T. cruzi-*exposed).

We further examined gene expression data using hierarchical clustering analysis of the DEGs. The clustering patterns correlate with the PCA observations, showing the genes with similar patterns of expression, and a strong contrast between 3D and 2D-cultured SYNs (Figure 1C, 3D vs. 2D, unexposed or *T. cruzi-*exposed). When we compared 3D vs. 2D SYNs cultures unexposed to *T. cruzi* (Figure 1D), we found 7,323 DEGs (4,334 upregulated genes and 2,989 downregulated genes). After *T. cruzi* exposure the number of DEGs increased to 8,318 genes, with 4,817 and 3,501 genes being up and downregulated, respectively.

**Figure 2:**
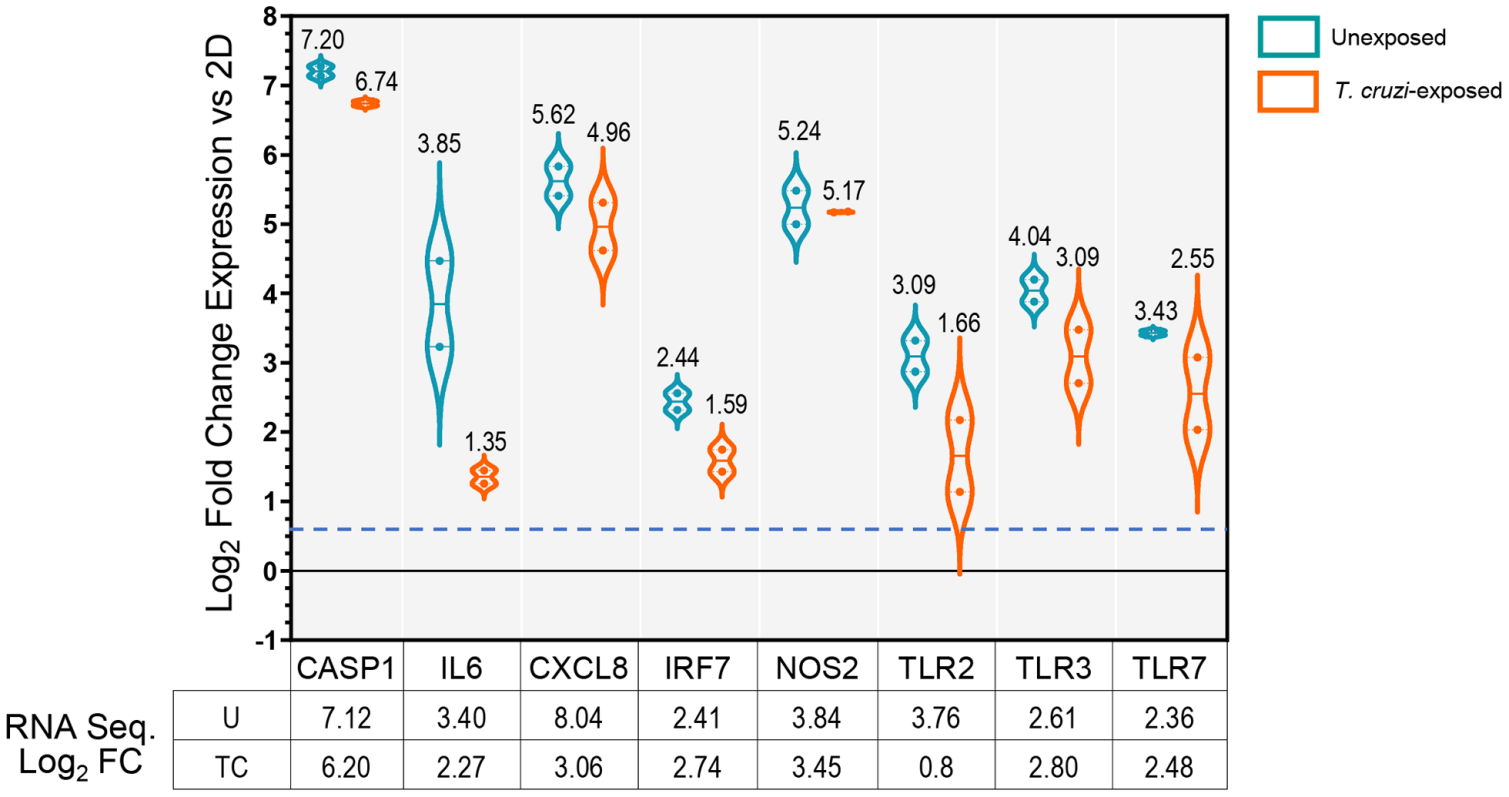
Validation of RNA-Seq data by qRT-PCR. mRNA quantification of selected genes carried out by qRT-PCR in unexposed or *T. cruzi*- exposed 3D SYNs. Violin-plot distributions showing log_2_ fold changes in gene expression in 3D SYNs compared to 2D SYNs as determined by the ∆C_t_ method with samples normalized to GAPDH. Dotted lines represent first and third quartiles, and solid lines indicate the median (values shown over plots). The dashed blue line indicates the assay cut off value (0.58). The qRT-PCR experiment was repeated two times with similar results. The table shows the log_2_ fold change in expression of 3D SYNs vs. 2D SYNs obtained through RNA sequencing analysis. FC: Fold change expression; U: unexposed; TC: exposed to *T. cruzi*.

To confirm the RNA-Seq data observations, we profiled the expression of selected genes by quantitative RT-PCR using arrays targeting molecules associated with host immune defenses (Figure 2). We observed upregulation of CASP1, IL6, CXCL8, IRF7, NOS2, TLR2, TLR3 and TLR7, which validates our whole transcriptome analysis findings. However, transcriptional levels of these genes remained nearly unchanged after exposure to *T. cruzi*. These data suggest that 3D SYNs constitutively express high levels of molecules involved in the activation of innate immune responses and correlate with the capacity of placental trophoblasts to control microbial infections (Ander et al., 2019; Megli et al., 2021; Semmes and Coyne, 2022).

### 3.2. Innate immune response signaling pathways are constitutively activated in 3D-cultured SYNs

To characterize 3D SYNs responses following *T. cruzi* exposure, we analyzed the significant changes in gene expression using Ingenuity Pathway Analysis (IPA).

**Figure 3:**
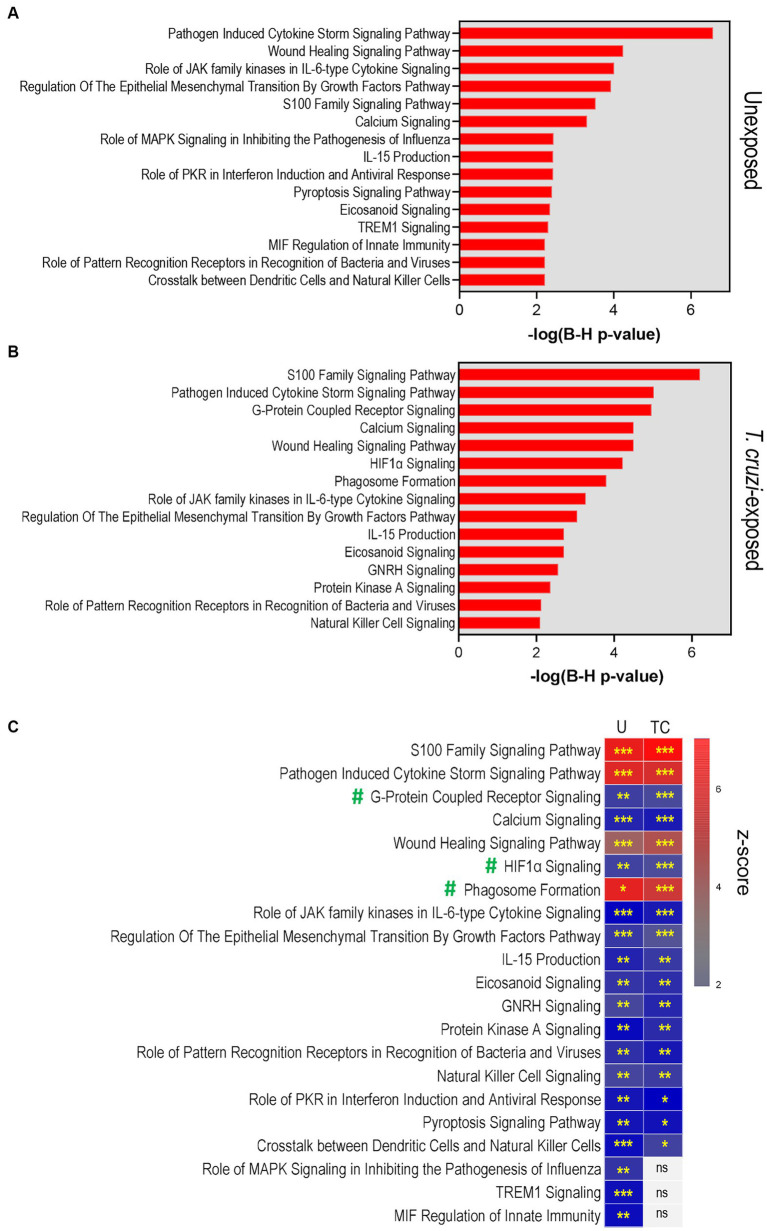
Pathway analysis reveals activation of processes associated with cellular immune defenses, pathogen-influenced signaling and cellular stress/injury in 3D-cultured SYNs. **(A)** Top 15 significant canonical pathways identified by IPA in 3D SYNs compared to 2D SYNs in cultures not exposed to *T. cruzi*. **(B)** Top significant canonical pathways identified by IPA in 3D SYNs compared to 2D SYNs in *T. cruzi*-exposed cultures. The red-colored bars represent the -log (B-H *p*-value) calculated for each pathway. **(C)** Heat-map showing the activation z-scores of the top canonical pathways in unexposed and *T. cruzi*-exposed cultures. Asterisks indicate the significance of each pathway comparing 3D and 2D SYNs (* B-H *p*-value <0.05; ** B-H *p*-value <0.01; *** B-H *p*-value <0.001). Only pathways with z-score ≥ 2 and B-H *p*-value ≥0.05 were considered significant. U: unexposed; TC: *T. cruzi*-exposed. ns: no significant B-H *p*-values. #: Pathways enriched in parasite-exposed 3D SYNs compared to 2D SYNs.

We first evaluated the canonical pathways activated in cultures that were not exposed to *T. cruzi*, comparing 3D SYNs to 2D SYNs (Figure 3A). The top 15 more significantly activated pathways included pathogen induced cytokine storm, wound healing, role of JAK family kinases in IL-6 type cytokine signaling, regulation of the epithelial mesenchymal transition by growth factors, S100 protein family, calcium signaling and role of MAPK signaling in inhibiting the pathogenesis of Influenza. Consistent with previous reports (Ander et al., 2019; Semmes and Coyne, 2022), our findings indicate that 3D SYNs not only constitutively express molecules associated with innate immunity, but they also exhibit wound repair and calcium-dependent signal transduction activities.

When we analyzed the top canonical pathways enriched in cultures after *T. cruzi* exposure (comparing 3D SYNs to 2D SYNs, Figure 3B), we observed activation of additional pathways such as G-protein coupled receptor signaling, HIF1α signaling and phagosome formation. Of note, S100 protein family signaling was the most significantly activated pathway followed by pathogen induced cytokine storm, calcium signaling and wound healing.

We next conducted a comparison analysis between unexposed and *T. cruzi*-exposed cultures to determine if there were differences in the z-scores and significance values (B-H *p*-values) between the top enriched pathways (Figure 3C; Supplementary Table 1). Based on the statistical analysis, pathways such as S100 protein family, pathogen induced cytokine storm, calcium signaling, and wound healing showed similar activation when cultures were unexposed or exposed to *T. cruzi*. However, G-protein coupled receptor signaling, HIF1α signaling and phagosome formation pathways were highly enriched in parasite-exposed 3D SYNs compared to 2D SYNs.

To initiate the culture of human SYNs in 3D, trophoblasts are co-cultured with human brain microvascular endothelial cells (HBMECs) attached to microcarrier beads. It was previously demonstrated that 3D-grown SYNs induce the dissociation of HBMECs from beads (McConkey et al., 2016) and form a layer that coats the surface of the spheroids (McConkey et al., 2016; Silberstein et al., 2021). Since some HBMECs may perhaps remain attached to beads after 20 days in culture, we also carried out RNA Seq analysis of HBMEC cultures grown in 3D. Next, we run an IPA core analysis to determine the canonical pathways activated in 2D SYNs compared to 3D HBMECs, unexposed or *T. cruzi-*exposed. Importantly, a comparison analysis of z-scores across all groups (3D vs. 2D SYNs and 3D HBMECs vs. 2D SYNs, Supplementary Figure 1) showed that the 3D SYNs top enriched canonical pathways where downregulated or not significant in 3D-grown HBMECs. Therefore, it’s unlikely that the presence of some residual HBMECs RNA in the 3D SYNs RNA preparation may possibly impact our transcriptome analysis results.

### 3.3. *Trypanosoma cruzi* modulates the gene expression landscape of 3D-cultured SYNs

**Figure 4:**
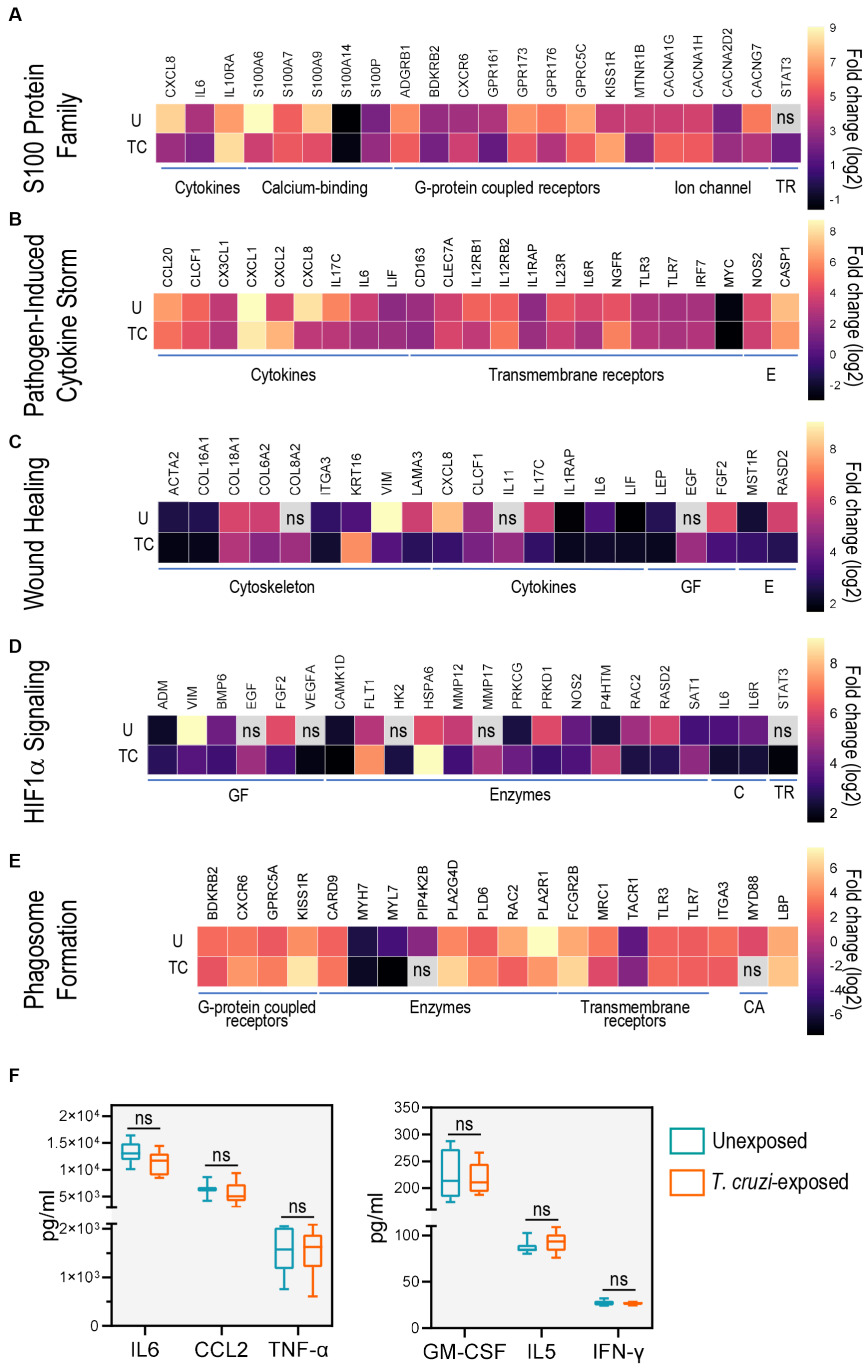
Toll-like receptors and cytokines are common genes in the top activated canonical pathways. Heat-maps of top differentially expressed genes for **(A)** S100 family, **(B)** pathogen induced cytokine storm, **(C)** wound healing, **(D)** HIF1α signaling and **(E)** phagosome formation pathways. Only genes with log_2_ fold change ≥ ± 1.5 and *p*-value ≥0.05 were considered significant. ns: no significant log_2_ fold change or B-H *p*-values. U: unexposed; TC: *T. cruzi*-exposed. **(F)** Levels of IL6, CCL2, TNF-α, GMCSF and IFN-γ determined using a Bio-Plex Pro Human Cytokine Screening Panel in culture supernatants of unexposed (blue) and *T. cruzi*-exposed (red) 3D-grown SYNs. Boxes represent first and third quartiles with a line at the median. Whiskers indicate maximum and minimum data values. CA: cytosolic adapter protein; C: cytokines and cytokine receptors; E: enzymes; GF: growth factors; TR: transcription regulators.

To better understand the different processes triggered in 3D SYNs after exposure to *T. cruzi*, we generated heatmaps of the most significantly regulated DEGs (log_2_ fold change ≥ ± 1.5; *p*-value <0.05) in the top enriched canonical pathways (Figure 4; Supplementary Figure 2; Supplementary Tables 2–6).

We first examined the gene expression patterns of molecules belonging to the S100 family signaling pathway, a family of calcium-binding proteins that are activators of immune functions including inflammation and also play an important role in calcium homeostasis (Xia et al., 2017; Singh and Ali, 2022). Of note, *T. cruzi* internalization is reported to be a Ca^2+^ dependent process (Fernandes and Andrews, 2012; Ferri and Edreira, 2021). We found that the most up-regulated molecules were CXCR6 and KISS1R (G-protein coupled receptors), IL10RA (cytokine receptor) and STAT3 (transcription regulator) while genes involved in cytoskeleton assembly (S100A6 and S100A9), calcium transport (CACNG7) and CXCL8 (cytokine) were down-regulated (Figure 4A; Supplementary Figure 2A; Supplementary Table 2).

Next, we studied the genes that were present in our data sets which are associated with pathogen induced cytokine storm signaling, a process characterized by uncontrolled inflammation caused by cytokine excess (Karki and Kanneganti, 2021). We observed up-regulation of CXCL2 (chemokine), and NGFR (TNF receptor). Expression of IL17C (cytokine) was down-regulated whereas transcription levels of other innate immunity-related molecules and factors (TLR3, TLR7, IRF7, NOS2, CASP1) only showed modest changes (Figure 4B; Supplementary Figure 2B; Supplementary Table 3).

We also found changes in the expression of several genes associated with the wound healing pathway, an important process required for *T. cruzi* internalization into host cells (Ferri and Edreira, 2021). Specifically, KRT16 and COL8A2 (structural proteins), EGF (growth factor), and IL11 (cytokine) were upregulated. In addition, a marked down-regulation of structural constituents VIM and LAMA3 were observed following *T. cruzi* exposure (Figure 4C; Supplementary Figure 2C; Supplementary Table 4).

Consistent with previous findings in human cardiomyocytes (Venturini et al., 2023), HIF1α signaling was also activated in 3D-grown SYNs exposed to the parasite. Transcripts encoding FLT1 (growth factor receptor), VEGFA (growth factor), HSPA6 (heat shock protein), HK2 (hexokinase), MMP17 (peptidase) and PRKCG (kinase) were the most enriched. PRKD1 (kinase) and RAC2 (GTPase) were among the most down-regulated genes (Figure 4D; Supplementary Figure 2D; Supplementary Table 5).

Phagocytosis is a fundamental process in immunity against pathogens (Rosales and Uribe-Querol, 2017) and *T. cruzi* exploits this mechanism to facilitate host cell invasion (Fernandes and Andrews, 2012; Ferri and Edreira, 2021). In the phagosome formation pathway, we identified genes such as GPRC5A (G-protein coupled receptor), FCGR2B (Fc receptor), PLA2G4D and PLD6 (phospholipases), LBP (lipopolysaccharide-binding protein) and CARD9 (caspase adaptor protein) whose transcript levels were greater in *T. cruzi*-exposed 3D SYNs than in unexposed cultures. We also found down-regulation of BDKRB2(G-protein coupled receptor), ITGA3, MRC1 and PLA2R1 (transmembrane receptors), and MYL7(calcium-binding protein) (Figure 4E; Supplementary Figure 2E; Supplementary Table 6).

To determine the commonly expressed genes across the top predicted canonical pathways we compared the lists generated by the IPA core analysis using a multiple list comparator tool^2^ (Supplementary Tables 6, 7). Among the genes shared by two or more pathways, we found molecules that are known to be involved in the innate immunity response to *T. cruzi* (Macaluso et al., 2023) such as TLR3 and TLR7 (shared by the pathogen induced cytokine storm and phagosome formation signaling pathways) and NOS2, STA3 and IL6 (shared by the S100 family, pathogen induced cytokine storm and HIF1α signaling pathways).

To further study the immunological profile of 3D SYNs, we measured cytokine and chemokine levels in culture supernatants using a multianalyte screening panel. In agreement with previous reports (Ander et al., 2018; Megli et al., 2021), we found that high levels (>1,000 pg/mL) of IL6, CCL2 and TNF-α were released to the culture media (Figure 4F, left panel). GM-CSF, IL5 and IFN-γ were also secreted but at lower levels (< 300 pg/mL; Figure 4F, right panel). Notably, constitutive expression of these molecules was not significantly altered following exposure to *T. cruzi*.

Together, the findings described above (Figures 3 4) strongly suggest that exposure of 3D SYNs to *T. cruzi* leads to activation of processes related to parasite sensing, internalization and phagocytosis (Fernandes and Andrews, 2012; Ferri and Edreira, 2021).

### 3.4. Blockade of TLR2 boosts *T. cruzi* growth

Toll-like receptors play an important role in the elimination of microbes through recognition of pathogen-associated patterns and induction of inflammatory cytokines and type I interferons (Fitzgerald and Kagan, 2020; Motomura et al., 2023). *T. cruzi* molecules such as surface glycoinositolphospholipids (GPIs) and parasite DNA/RNA sequences can be sensed by TLRs (Macaluso et al., 2023). In fact, several investigators have shown the significance and involvement of TLRs in the control of *T. cruzi* infection (Caetano et al., 2011; Castillo et al., 2017b; Queiroga et al., 2021; Ait Djebbara et al., 2023).

Because we found that molecules associated with the TLR2 pathway (IL6, CXCL8 and CXCL2), and TLR3 and TLR7 were differentially regulated in 3D SYNs compared to 2D SYNs (Figure 4; Supplementary Figure 2), we explored their function as mediators of trophoblasts’ immunity to *T. cruzi*. For these experiments, we could not employ 3D SYNs because they resist parasite infection (Silberstein et al., 2021). Therefore, we used 2D SYNs as working model since they not only express TLRs (although to lower levels compared to 3D SYNs) but also support *T. cruzi* growth.

**Figure 5:**
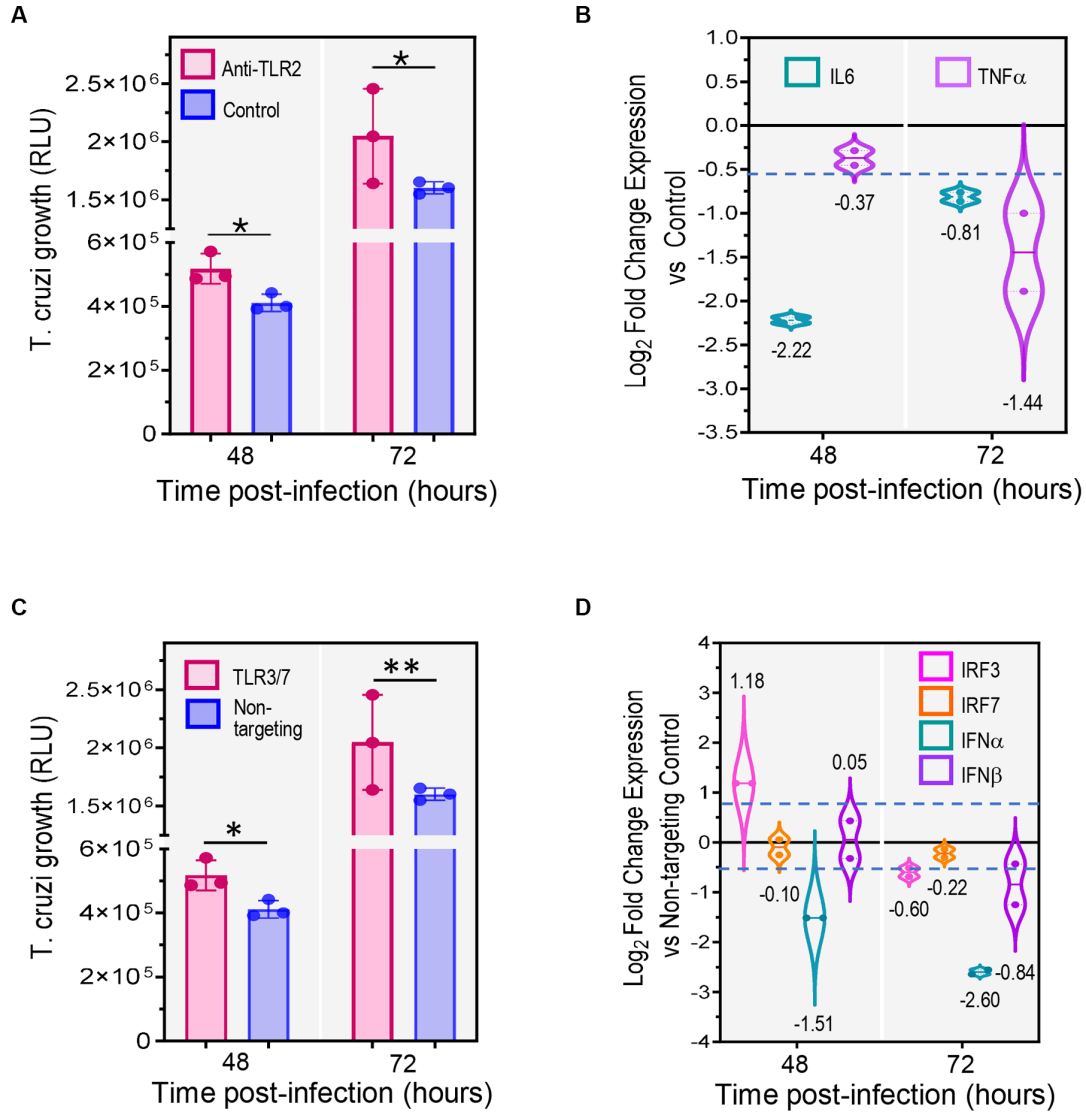
Inhibition of TLRs significantly decreases expression of cytokines and interferons promoting *T. cruzi* growth. 2D SYNs were treated with anti-hTLR2 (TLR2 blockade) or transfected with TLR3/TLR7 siRNA mix, followed by *T. cruzi* infection at ratio of 5 parasites/cell. **(A)** *T. cruzi* growth over time after TLR2 blockade or **(C)** TLR3/TLR7 siRNA transfection. Bars show the nanoluciferase activity expressed in relative luminescence units (RLU) measured at 48 and, 72 hpi and represent the mean ± SD of three technical replicates. The experiment was repeated two times with similar results. Asterisks indicate statistically significant differences calculated using an unpaired *t*-test. *: *p* ≤ 0.05; **: *p* ≤ 0.01. **(B)** qRT-PCR mRNA quantification after TLR2 blockade or **(D)** TLR3/TLR7 siRNA transfection. Fold changes in gene expression compared to cells treated with isotype control antibodies **(B)** or to cells transfected with non-targeting siRNA control **(D)**, were determined by the ∆C_t_ method with samples normalized to GAPDH. Violin-plots show the log_2_ fold change expression vs. control (mean ± SD) of two technical replicates. Dotted lines represent first and third quartiles, and solid lines indicate the median (values shown below or above the plots). The dashed blue line shows the assay cut off value (0.58). The experiment was repeated two times with similar results.

To study the effect of TLR2 blockade on parasite infection, 2D SYNs were pre-treated with anti-hTLR2 antibodies or isotype control antibody (5 μg/mL each) and next infected with TcCOL-NLuc trypomastigotes. Parasite growth was monitored at 48 and 72 h post infection (hpi) (Figure 5A) by measuring nanoluciferase activity as described previously (Silberstein et al., 2021). We found that TLR2 inhibition significantly promoted parasite growth as indicated by the increase in nanoluciferase activity in cells treated with anti-TLR2 antibody at both 48 and 72 hpi. Since TLR2 activation triggers expression of IL6 and TNF-α, we explored the downstream effects of TLR2 inhibition by measuring their mRNA levels by qRT-PCR (Figure 5B). The most reduced cytokine in anti-TLR2 antibody treated 2D SYNs was IL6 (fourfold down-regulation), at 48 hpi while a marked decrease in TNF-α was found at 72 hpi.

### 3.5. TLR3 and TLR7 siRNA silencing stimulates *T. cruzi* expansion

We also investigated the outcome of TL3 and TLR7 double knockdown on *T. cruzi* infection. To test this, 2D SYNs were transfected with TLR3/TLR7 siRNAs or non-targeting siRNA (control). Gene silencing was verified by measuring TLR3 and TLR7 mRNA quantities using qRT-PCR (Supplementary Figure 3). Twenty-four hours post-transfection, cells were infected with TcCOL-NLuc trypomastigotes and parasite multiplication was monitored as explained above. We found that knockdown of TLR3 and TLR7 also promoted parasite growth as evidenced by the significant increase in nanoluciferase activity in double knock-downed cells (Figure 5C). Given that TLR3 and TLR7 downstream signaling involves activation of interferon regulatory factors (IRFs), which in turn trigger synthesis of type I interferon genes, we measured IRF3, IRF7, IFN-α and IFN-β mRNA levels by qRT-PCR (Figure 5D). The knockdown effect on cytokine mRNAs expression was observed for IRF3, IFN-α and IFN-β at 72 h post-transfection, with a remarkable 2.5-fold reduction in IFN-α.

These data (Figure 5) suggest that inhibition of TLR2, TLR3 and TLR7 causes a reduction in the expression of immunoregulatory molecules, which results in increased parasite growth. Furthermore, our findings highlight the significant role played by TLRs in SYNs’ innate response and resistance to *T. cruzi* infection.

## 4. Discussion

Congenital *T. cruzi* infection is now the main route of CD transmission in non-endemic countries where pregnant women are not routinely tested for the disease (Antinori et al., 2017). A complex parasite–host cell interplay occurs at the maternal-fetal interface when bloodstream trypomastigotes interact with the SYNs (Blaszkowska and Goralska, 2014). Understanding the molecular basis of *T. cruzi* invasion and replication in SYNs could provide insights into the specific molecules and host signaling pathways that modulate parasite transmission from mother-to-fetus.

Although composed by different trophoblasts populations (SYNs, cytotrophoblast, and extravillous trophoblasts), maternal macrophages and Hofbauer cells (Semmes and Coyne, 2022), human chorionic villi explants have been previously used to examine the transcriptome changes induced after *T. cruzi* challenge (Castillo et al., 2018). However, the signaling pathways activated in monotypic cultures of SYNs in the context of parasite infection, have not been investigated. Using 3D-grown cultures, and through RNA sequencing and whole transcriptome analysis, we characterized the SYN’s response to *T. cruzi* and confirmed their key role as immune sensors of parasite infection.

Consistent with previous work, our data revealed significant activation of signaling pathways associated with the innate immune response (Castillo et al., 2018; Barbosa et al., 2022). Notably, we observed that pathways such as S100 protein family, pathogen induced cytokine storm, calcium signaling, and wound healing were similarly enriched in unexposed or *T. cruzi* exposed 3D SYNs compared to 2D SYNs (Figure 3C). Indeed, induction of these canonical pathways was verified through the detection of cytokines and chemokines (Figure 4F) in the culture media, as reported by others (Vekemans et al., 2000; Arora et al., 2017; Corry et al., 2017; Ander et al., 2019). We also found overexpression of caspase 1 (CASP1, Figures 2, 4B), a key driver of placental antimicrobial defenses through the inflammasome signaling pathway (Megli et al., 2021). These findings suggest that 3D SYNs exhibit a pro-inflammatory state that shapes their resistance to *T. cruzi* and other microbial infections.

During its life cycle inside the host-cell, *T. cruzi* hijacks different cell functions to establish a productive infection. The main steps of parasite invasion include host cell recognition and adhesion, internalization, formation of and escape of parasites from the phagolysosome, multiplication of amastigotes in the cytosol where they transform into trypomastigotes that can infect new cells (Ferri and Edreira, 2021). Based on our data, it is likely that 3D SYNs resist *T. cruzi* infection by modulating the expression of host molecules linked to the top canonical pathways identified in this work. These proteins may play a role in inhibiting the parasite’s intracellular cycle at different stages.

**Figure 6:**
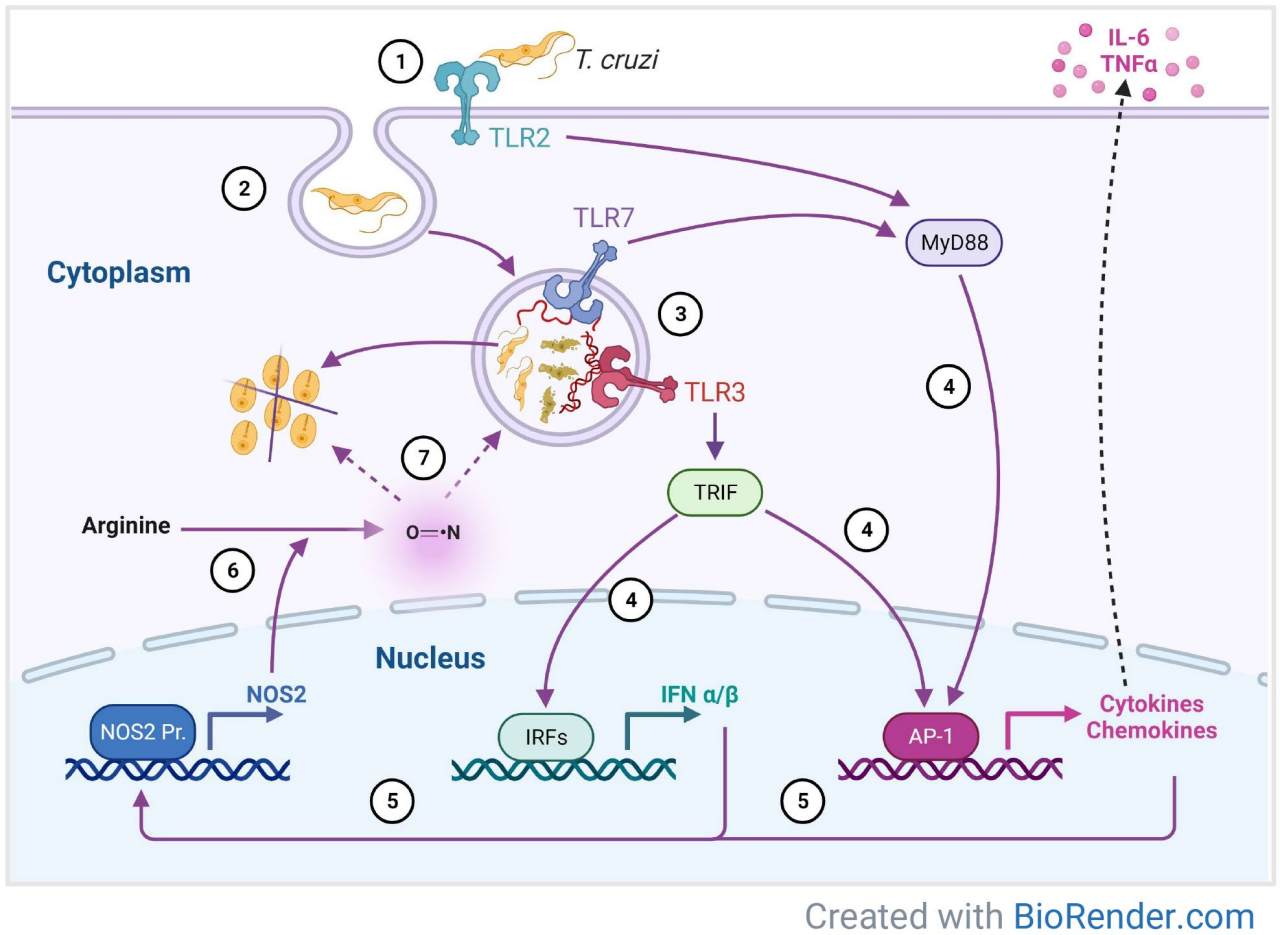
A proposed model of immune response mechanisms against *T. cruzi* mediated by Toll-like receptors in 3D-grown SYNs. *T. cruzi* invasion begins with binding to the SYNs extracellular matrix followed by TLR2 recognition and adhesion to the cell (1). This interaction promotes parasite internalization (2) into the parasitophorous vacuole and subsequent formation of the phagolysosome, where DNA and RNA digestion products are recognized by TLR3 and 7 (3). Parasite sensing by TLRs leads to synthesis of cytokines, chemokines and INFα/β (4), which in turn induce expression of NOS2 (5). Oxidation of arginine by NOS2 (6) generates nitric oxide which kills the parasite inside the phagolysosome or in the cytosol (7).

For instance, *T. cruzi* can be recognized by cell surface TLRs and some G protein coupled receptor such as bradykinin B2 (BDKRB2), which in turn trigger innate defense mechanisms in the host cell (Schmitz et al., 2014; Castillo et al., 2017b; Macaluso et al., 2023). Notably, we observed up-regulation of TLR2 (Figure 2), and BDKRB2 (Figures 4A,E; associated with the S100 protein family pathway) in 3D SYNs. Blockade of TLR2 resulted in more *T. cruzi* replication (Figure 5A) likely due to a reduction in TLR2-mediated signaling and cytokines production (Figure 6). Interestingly, we have not detected significant overexpression of TLR4, reported to induce IFN-γ and TNF-α production in human cord blood cells after stimulation a recombinant *T. cruzi* macrophage infectivity potentiator (TcMIP) (Ait Djebbara et al., 2023). Likewise, we have not observed an increase in the level of nuclear factor kappa B (NF-κB) RNA transcripts, a molecule shown to be activated in placental explants *via* TLRs signaling pathways (Liempi et al., 2019). However, we detected significant up-regulation of the AP-1 transcription subunits FOSL1 and JUN in unexposed (log_2_ FC 3.5 and 4.2, respectively) and *T. cruzi*-exposed (log_2_ FC 3.6 and 4.2, respectively) SYNs (Supplementary Table 9). Activation of AP-1 leads to production of proinflammatory cytokines such us IL6 and TNF-α (Duan et al., 2022) and could explain the detection of these cytokines in supernatants of *T. cruzi* exposed SYNs (Figure 4F).

Another crucial event required for *T. cruzi* cell invasion is the mobilization of calcium (Ca^2+^) for lysosome recruitment. When trypomastigotes wound host cells to access the cytoplasm, they trigger Ca^2+^ influx from intracellular stores and the extracellular environment (Fernandes and Andrews, 2012; Ferri and Edreira, 2021). In agreement with former studies that evaluated the alterations of gene expression in placentas of pregnant women with chronic CD (Juiz et al., 2018), we found enrichment of the calcium signaling pathway (Figure 3). In addition, and as mentioned above, we also observed activation of the S100 protein family pathway, which includes Ca^2+^ binding and Ca^2+^ voltage-gated channel molecules. S100 proteins regulate diverse cellular processes including intracellular Ca^2+^ homeostasis (Sreejit et al., 2020) and their expression may be associated with HBV intrauterine transmission (Zhao et al., 2020). It is possible that SYNs alter their Ca^2+^ intracellular storage and mobilization limiting parasite invasion. However, the specific Ca^2+^ binding and transport proteins along with their role in the context of placenta infection by *T. cruzi*, remain to be investigated.

In line with the capacity of SYNs to experience a continuous and dynamic repair process (Castillo et al., 2018; Hoo et al., 2020), we found that the wound healing signaling pathway was significantly enriched in 3D SYNs. Because damage of the host cell plasma membrane is required for parasite entry (Brigido et al., 2017), our results suggest that SYNs’ constitutive healing mechanism can be actively inhibiting this important process. Additionally, we detected down-regulation of the expression of laminin-subunit alpha 3 (LAMA3) and vimentin (VIM) along with a marked up-regulation of cytokeratin 16 (KRT16) (Figure 4C; Supplementary Figure 2C). It’s been shown that Tc-85 proteins (members of the gp85/trans-sialidase superfamily) are involved in cell adhesion and parasite cell entry by interaction with molecules such as laminin (extracellular matrix constituent) and the intermediate filaments proteins cytokeratin and vimentin (Teixeira et al., 2015). Thus, downregulation of LAMA3 and VIM may possibly constitute another mechanism by which SYNs restrict parasite colonization while KRT16 over expression could be needed to maintain cell shape and integrity.

*T. cruzi* exploits several internalization pathways (including phagocytosis) for cell invasion (Ferri and Edreira, 2021). As reviewed by Rosales et al. (Rosales and Uribe-Querol, 2017), phagocytosis is a fundamental process in immunity which concludes with the formation of phagolysosomes where parasites are eventually digested and killed (Cardoso et al., 2015). In fact, we found significant activation of the phagosome formation pathway following exposure to the parasite (Figures 3B,C). We also observed upregulation of phospholipases PLD6 and PLA2G4D, enzymes known to be expressed in phagocytes (Griffiths and Mayorga, 2007; Dabral and van den Bogaart, 2021) and TLR3 and 7, pattern recognition receptors that detect nucleic acids from lysed parasites in the phagolysosome (Cardoso et al., 2015). Furthermore, we show that silencing of TLR3 and 7 increased *T. cruzi* infection (Figure 5C). Surprisingly, we have not found significant overexpression of TLR9 in 3D SYNs compared to 2D SYNs although it was reported that *T. cruzi* can also be sensed by intracellular TLR9 (Bafica et al., 2006; Gravina et al., 2013). Together, these findings indicate that altering phagocytosis-related pathways, including increased expression of TLR3 and 7, could be one more strategy to control *T. cruzi* colonization of 3D SYNs.

Previous reviews have explored the crosstalk between hypoxia-inducible factor (HIF-1α) and nitric oxide (NO) signaling pathways (Olson and van der Vliet, 2011; Pappas et al., 2023). Besides inducing the expression of proteins implicated in glucose metabolism such as HK2, HIF-1α can activate NOS2 and trigger NO production. Consistent with recent reports, our transcriptome analysis studies unveiled activation of the HIF-1α pathway (Figures 3B,C) along with overexpression of NOS2 (Figures 2 4D; Supplementary Figure 2D) and HK2 (Figure 4D; Supplementary Figure 2D) in parasite-exposed 3D SYNs (Triquell et al., 2018; Silberstein et al., 2021; Venturini et al., 2023). Upregulation of NOS2 may increase arginine oxidation which in turn could generate high NO levels to kill *T. cruzi* (Cardoso et al., 2015). Our results suggest that activation of the HIF-1α pathway by SYNs could also contribute to their resistance to *T. cruzi*.

It is important to note that even though 3D SYNs have characteristics similar to human syncytiotrophoblasts, including the ability to resist microbial infections (McConkey et al., 2016; Corry et al., 2017; Silberstein et al., 2021), this cell-based system may not completely reproduce *in vivo* responses to *T. cruzi* infection. In fact, the maternal-fetal interface is a complex environment where the chorionic villi are associated with the decidua containing different immune cell populations which can also impact immunity signaling pathways in SYNs (Semmes and Coyne, 2022). While SYNs have been reported to be a route of parasite transmission to fetal tissues, it has also been shown that *T. cruzi* invasion can occur through infection of non-trophoblastic epithelial cells located at the placenta marginal sinus (Fernandez-Aguilar et al., 2005; Carlier et al., 2020). Our 3D cell culture system addresses only the role of SYNs in the control of *T. cruzi* vertical transmission, and therefore our observations may specifically apply to these trophoblastic cell type. Another limitation of this work is that the functional analysis experiments only evaluated the role of TLRs in controlling parasite growth in the context of a low parasite exposure representing the scenario of a chronic infection in pregnant women. Further studies using higher parasite loads and focused on the functionality of the activated signaling pathways and molecules predicted by IPA, may provide additional insights into the multiple resistance processes that limit *T. cruzi* infection of SYNs.

Our data support a model by which 3D SYNs may resist infection by modulating TLR signaling pathways (Figure 6). *T. cruzi* invasion begins with parasite binding to laminin (LAMA3) within the extracellular matrix. Next, parasites are recognized by TLR2 and adhere to the cell. This interaction promotes internalization into the parasitophorous vacuole and subsequent formation of the phagolysosome where some parasites are digested by enzymes and others escape into the cytoplasm. The DNA and RNA digestion products are recognized by TLR3 and 7 inside the phagolysosome. Parasite sensing by TLRs leads to synthesis of cytokines, chemokines and INFα/β, which in turn induce expression of NOS2. Oxidation of arginine by NOS2 generates nitric oxide which could kill the parasite either inside the phagolysosome or in the cytosol. Thus, downregulation of laminin (LAMA3) and overexpression of TLRs and NOS2, could potentially impact parasite growth in SYNs at different stages of the parasite intracellular life cycle.

Collectively, our studies show that increased TLRs signaling and resulting inflammatory responses may together play an important role in SYNs’ resistance to *T. cruzi*. Understanding the immunological landscape of the placenta, including the contribution of SYNs and how innate immunity is regulated, may contribute to the development of new therapeutics to reduce the risk of congenital CD.

## Data availability statement

The datasets presented in this study can be found in online repositories. The names of the repository/repositories and accession number(s) can be found at: NCBI – PRJNA994533.

## Author contributions

ES: Conceptualization, Formal analysis, Investigation, Methodology, Validation, Visualization, Writing – original draft, Writing – review & editing. CC: Data curation, Formal analysis, Software, Writing – review & editing. AD: Conceptualization, Formal analysis, Supervision, Writing – review & editing.

## Funding

The author(s) declare financial support was received for the research, authorship, and/or publication of this article. This research was supported by the intramural grants from the U.S. Food and Drug Administration to AD. The funders had no role in study design, data collection and analysis, decision to publish, or preparation of the manuscript. The findings and conclusions in this article have not been formally disseminated by the Food and Drug Administration and should not be construed to represent any Agency determination and policy.

## Conflict of interest

The authors declare that the research was conducted in the absence of any commercial or financial relationships that could be construed as a potential conflict of interest.

## Publisher’s note

All claims expressed in this article are solely those of the authors and do not necessarily represent those of their affiliated organizations, or those of the publisher, the editors and the reviewers. Any product that may be evaluated in this article, or claim that may be made by its manufacturer, is not guaranteed or endorsed by the publisher.

## Footnotes

^1^ http://www.novogen.com

^2^ https://molbiotools.com/listcompare.php
