# Supplementary material for: The transcriptome landscape of 3D-cultured placental trophoblasts reveals activation of TLR2 and TLR3/7 in response to low Trypanosoma cruzi parasite exposure
Source: Front Microbiol. 2023 Sep 20;14:1256385. doi: 10.3389/fmicb.2023.1256385 (PMC10548471; doi:10.3389/fmicb.2023.1256385)
Supplement: Supplementary file 1 [file Data_Sheet_1.PDF]

Supplementary Tables

Supplementary Table 1: Top canonical pathways in unexposed and *T. cruzi*-exposed cultures predicted by Ingenuity Pathway Analysis.

Canonical Pathway	Unexposed		*T. cruzi*-exposed	
	z-score	B-H p-value	z-score	B-H p-value
S100 Family Signaling Pathway	6.70	3.20×10^{-4}	7.04	6.20×10^{-7}
Pathogen Induced Cytokine Storm Signaling Pathway	6.48	2.70×10^{-7}	6.35	9.50×10^{-6}
G-Protein Coupled Receptor Signaling	3.40	9.00×10^{-3}	3.65	1.10×10^{-5}
Calcium Signaling	2.78	4.90×10^{-4}	2.56	3.20×10^{-5}
Wound Healing Signaling Pathway	5.44	5.70×10^{-5}	5.71	3.20×10^{-5}
HIF1α Signaling	3.45	8.00×10^{-3}	3.73	6.30×10^{-5}
Phagosome Formation	6.6	1.20×10^{-2}	6.18	1.60×10^{-4}
Role of JAK family kinases in IL-6-type Cytokine Signaling	2.04	9.80×10^{-5}	2.56	6.30×10^{-4}
Regulation of the Epithelial Mesenchymal Transition by Growth Factors Pathway	3.24	1.12×10^{-4}	3.89	8.90×10^{-4}
IL-15 Production	2.75	4.00×10^{-3}	3.27	2.00×10^{-3}
Eicosanoid Signaling	3.16	4.00×10^{-3}	3.00	2.00×10^{-3}
GNRH Signaling	3.58	1.00×10^{-2}	2.84	3.00×10^{-3}
Protein Kinase A Signaling	2.14	9.00×10^{-3}	2.94	6.00×10^{-3}
Role of Pattern Recognition Receptors in Recognition of Bacteria and Viruses	3.05	6.00×10^{-3}	2.50	4.00×10^{-3}
Natural Killer Cell Signaling	3.55	1.00×10^{-2}	3.33	8.00×10^{-3}
Role of PKR in Interferon Induction and Antiviral Response	2.43	4.00×10^{-3}	1.96	1.40×10^{-2}
Pyroptosis Signaling Pathway	2.40	4.00×10^{-3}	2.36	4.80×10^{-2}
Crosstalk between Dendritic Cells and Natural Killer Cells	2.22	6.00×10^{-3}	3.46	4.20×10^{-2}
Role of MAPK Signaling in Inhibiting the Pathogenesis of Influenza	3.13	4.00×10^{-3}	2.84	ns
TREM1 Signaling	2.30	5.00×10^{-3}	3.74	ns
MIF Regulation of Innate Immunity	2.22	6.00×10^{-3}	2.33	ns

ns: no significant B-H p-values

Supplementary Table 2: S100 Protein Family Signaling Pathway

Symbol	Gene Name	Unexposed		*T. cruzi*-exposed	
		Fold Change (log_2)	p-value	Fold Change (log_2)	p-value
**CXCL8**	C-X-C motif chemokine ligand 8	8.04	3.74 E-07	3.05	9.95E-06
**IL6**	Interleukin 6	3.42	1.94 E-03	2.27	2.35E-02
**IL10RA**	Interleukin 10 receptor subunit alpha	6.84	2.92×10^{-5}	8.29	9.60E-08
**S100A6**	S100 calcium binding protein A6	9.05	1.89×10^{-17}	4.25	2.10E-17
**S100A7**	S100 calcium binding protein A7	5.44	3.52×10^{-3}	5.28	5.82E-03
**S100A9**	S100 calcium binding protein A9	7.94	1.97×10^{-13}	4.88	4.81E-05
**S100A14**	S100 calcium binding protein A14	-1.61	9.56×10^{-12}	-1.27	4.18E-08
**S100P**	S100 calcium binding protein P	2.11	2.90×10^{-76}	2.87	1.30E-03
**ADGRB1**	Adhesion G protein-coupled receptor B1	6.44	1.11×10^{-4}	5.39	4.43E-97
**BDKRB2**	Bradykinin receptor B2	2.90	3.56×10^{-4}	2.00	9.94E-04
**CXCR6**	C-X-C motif chemokine receptor 6	3.19	1.60×10^{-24}	4.32	8.48E-32
**GPR161**	G protein-coupled receptor 161	3.50	2.43×10^{-6}	1.59	7.07E-03
**GPR173**	G protein-coupled receptor 173	6.62	7.1×10^{-5}	5.20	2.31E-03
**GPR176**	G protein-coupled receptor 176	5.96	5.69×10^{-4}	3.61	9.08E-03
**GPRC5C**	G protein-coupled receptor class C group 5 member C	7.04	6.46×10^{-6}	5.41	3.47E-14
**KISS1R**	KISS1 receptor	4.09	1.38×10^{-3}	6.96	2.18E-05
**MTNR1B**	Melatonin receptor 1B	4.20	6.83×10^{-24}	2.60	4.28E-11
**CACNA1G**	Calcium voltage-gated channel subunit alpha1 G	4.22	ns	5.51	1.19E-03
**CACNA1H**	Calcium voltage-gated channel subunit alpha1 H	4.42	7.87×10^{-6}	5.30	1.86E-08
**CACNA2D2**	Calcium voltage-gated channel auxiliary subunit alpha2delta 2	1.98	4.42×10^{-3}	2.97	1.03E-04
**CACNG7**	Calcium voltage-gated channel auxiliary subunit gamma 7	6.08	1.55×10^{-4}	3.66	2.54E-06
**STAT3**	Signal transducer and activator of transcription 3	ns	3.37E-65	1.77	6.05E-89

ns: no significant p-values

Supplementary Table 3: Pathogen Induced Cytokine Storm Signaling Pathway

Symbol	Gene Name	Unexposed		*T. cruzi*-exposed	
		Fold Change (log_2)	p-value	Fold Change (log_2)	p-value
**CCL20**	C-C motif chemokine ligand 20	6.32	1.83E-04	5.20	2.54E-03
**CLCF1**	Cardiotrophin like cytokine factor 1	4.88	4.92E-03	4.25	2.00E-02
**CX3CL1**	C-X3-C motif chemokine ligand 1	3.14	2.27E-06	2.75	2.41E-04
**CXCL1**	C-X-C motif chemokine ligand 1	8.74	1.70E-08	8.23	1.60E-07
**CXCL2**	C-X-C motif chemokine ligand 2	3.80	3.35E-04	6.89	4.04E-05
**CXCL8**	C-X-C motif chemokine ligand 8	8.04	3.74E-07	3.05	9.95E-06
**IL17C**	Interleukin 17C	5.71	1.38E-03	2.98	4.75E-02
**IL6**	Interleukin 6	3.42	0.00194	2.27	2.35E-02
**LIF**	LIF interleukin 6 family cytokine	1.67	5.51E-25	2.17	6.01E-35
**CD163**	CD163 molecule	2.15	7.19E-03	1.73	2.99 E-02
**CLEC7A**	C-type lectin domain containing 7A	3.55	5.05E-29	3.85	7.28E-28
**IL12RB1**	Interleukin 12 receptor subunit beta 1	4.86	1.26E-02	3.10	4.20E-02
**IL12RB2**	Interleukin 12 receptor subunit beta 2	4.77	1.71E-02	5.33	5.58E-03
**IL1RAP**	Interleukin 1 receptor accessory protein	1.72	2.84E-75	2.08	2.39E-110
**IL23R**	Interleukin 23 receptor	4.39	1.39E-02	3.35	2.00E-02
**IL6R**	Interleukin 6 receptor	3.90	1.93E-04	2.39	5.74E-04
**NGFR**	Nerve growth factor receptor	4.32	1.57E-02	5.65	2.04 E-06
**TLR3**	Toll like receptor 3	2.61	9.33E-19	2.84	4.30E-15
**TLR7**	Toll like receptor 7	2.36	2.04E-11	2.48	1.60E-08
**IRF7**	Interferon regulatory factor 7	2.41	2.01E-06	2.74	4.03E-08
**MYC**	MYC proto-oncogene, bHLH transcription factor	-2.69	3.31E-73	-3.00	3.51E-85
**NOS2**	Nitric oxide synthase 2	3.84	1.97E-04	3.45	6.07E-04
**CASP1**	Caspase 1	7.12	5.18E-06	6.18	1.34E-11

Supplementary Table 4: Wound Healing Signaling Pathway

Symbol	Gene Name	Unexposed		*T. cruzi*-exposed	
		Fold Change (log_2)	p-value	Fold Change (log_2)	p-value
**ACTA2**	Actin alpha 2, smooth muscle	2.57	1.12E-03	1.93	9.85E-03
**COL16A1**	Collagen type XVI alpha 1 chain	2.70	1.64E-10	2.01	1.38E-08
**COL18A1**	Collagen type XVIII alpha 1 chain	5.96	3.45E-55	5.31	3.18E-62
**COL6A2**	Collagen type VI alpha 1 chain	5.77	7.98E-32	4.51	1.08E-41
**COL8A2**	Collagen type VIII alpha 2 chain	4.27	ns	4.93	2.4E-02
**ITGA3**	Integrin subunit alpha 3	2.96	4.11E-157	2.32	2.98E-80
**KRT16**	Keratin 16	3.48	1.51E-03	7.30	7.14E-06
**VIM**	Vimentin	9.05	3.08E-16	3.57	4.26E-12
**LAMA3**	Laminin subunit alpha 3	5.74	1.11E-03	2.83	1.47E-02
**CXCL8**	C-X-C motif chemokine ligand 8	8.04	3.74E-07	3.05	9.95E-06
**CLCF1**	Cardiotrophin like cytokine factor 1	4.88	4.92E-03	4.25	2.00E-02
**IL11**	Interleukin 11	ns	ns	4.79	3.74E-02
**IL17C**	Interleukin 17C	5.71	1.38E-03	2.98	4.75E-02
**IL1RAP**	Interleukin 1 receptor accessory protein	1.72	2.84E-75	2.08	2.39E-110
**IL6**	Interleukin 6	3.42	0.00194	2.27	2.35E-02
**LIF**	LIF interleukin 6 family cytokine	1.67	5.51E-25	2.17	6.01E-35
**LEP**	Leptin	2.76	3.27E-04	2.09	2.83E-03
**EGF**	Epidermal growth factor	3.88	ns	4.89	1.34E-02
**FGF2**	Fibroblast growth factor 2	6.13	3.22E-04	3.38	1.78E-02
**MST1R**	Macrophage stimulating 1 receptor	2.38	4.83E-07	3.07	1.55E-07
**RASD2**	RASD family member 2	5.86	9.47E-04	2.75	2.06E-02

ns: no significant p-values

Supplementary Table 5: HIF1α Signaling Pathway

Symbol	Gene Name	Unexposed		*T. cruzi*-exposed	
		Fold Change (log_2)	p-value	Fold Change (log_2)	p-value
**ADM**	Adrenomedullin	2.17	1.90E-99	2.81	2.01E-11
**VIM**	Vimentin	9.05	3.08E-16	3.57	4.26E-12
**BMP6**	Bone morphogenetic protein 6	4.02	3.07E-02	3.16	7.57E-03
**EGF**	Epidermal growth factor	3.88	ns	4.89	1.34E-02
**FGF2**	Fibroblast growth factor 2	6.13	3.22E-04	3.38	1.78E-02
**VEGFA**	Vascular endothelial growth factor A	ns	2.36E-48	1.88	6.41E-49
**CAMK1D**	Calcium/calmodulin dependent protein kinase ID	2.25	1.12E-03	1.63	5.12E-03
**FLT1**	Fms related receptor tyrosine kinase 1	5.33	3.45E-06	7.33	2.38E-06
**HK2**	hexokinase 2	ns	3.99E-05	2.51	1.70E-14
**HSPA6**	heat shock protein family A (Hsp70) member 6	6.06	4.08E-07	9.02	1.96E-06
**MMP12**	matrix metallopeptidase 12	5.60	1.86E-03	3.20	6.20E-03
**MMP17**	matrix metallopeptidase 17	ns	ns	5.12	2.64E-03
**PRKCG**	protein kinase C gamma	2.50	3.15E-02	3.96	2.31E-03
**PRKD1**	protein kinase D1	6.06	4.41E-04	3.09	9.09E-03
**NOS2**	Nitric oxide synthase 2	3.84	1.97E-04	3.45	6.07E-04
**P4HTM**	prolyl 4-hydroxylase, transmembrane	2.47	6.10E-03	5.68	7.24E-04
**RAC2**	Rac family small GTPase 2	4.94	3.85E-03	2.52	9.78E-05
**RASD2**	RASD family member 2	5.86	9.47E-04	2.75	2.06E-02
**IL6**	Interleukin 6	3.42	0.00194	2.27	2.35E-02
**IL6R**	Interleukin 6 receptor	3.90	1.93E-04	2.39	5.74E-04
**SAT1**	Spermidine/spermine N1-acetyltransferase 1	3.26	2.42E-28	4.63	3.79E-43
**STAT3**	signal transducer and activator of transcription 3	ns	3.37E-65	1.77	6.05E-89

ns: no significant p-values

Supplementary Table 6: Phagosome Formation Signaling Pathway

Symbol	Gene Name	Unexposed		*T. cruzi*-exposed	
		Fold Change (log_2)	p-value	Fold Change (log_2)	p-value
**BDKRB2**	Bradykinin receptor B2	2.90	3.56×10^{-4}	2.00	9.94E-04
**CXCR6**	C-X-C motif chemokine receptor 6	3.19	1.60×10^{-24}	4.32	8.48E-32
**GPRC5A**	G protein-coupled receptor class C group 5 member A	2.255	1.68E-04	3.513	9.47E-10
**KISS1R**	KISS1 receptor	4.09	1.38×10^{-3}	6.96	2.18E-05
**CARD9**	Caspase recruitment domain family member 9	2.593	2.78E-10	3.246	5.65E-12
**MYH7**	Myosin heavy chain 7	-5.768	2.42E-07	-6.675	1.97E-20
**MYL7**	Myosin light chain 7	-4.272	4.51E-06	-7.679	9.94E-17
**PIP4K2B**	Phosphatidylinositol-5-phosphate 4-kinase type 2 beta	-1.641	3.47E-03	-0.466	5.95E-01
**PLA2G4D**	Phospholipase A2 group IVD	3.824	4.36E-02	6.319	2.70E-04
**PLD6**	Phospholipase D family member 6	2.421	3.73E-02	3.969	2.33E-03
**RAC2**	Rac family small GTPase 2	4.938	3.85E-03	2.516	9.78E-05
**PLA2R1**	Phospholipase A2 receptor 1	7.719	1.25E-06	4.322	2.14E-05
**FCGR2B**	Fc gamma receptor IIb	4.907	1.14E-02	6.379	1.70E-04
**MRC1**	Mannose receptor C-type 1	3.315	1.31E-04	1.502	4.45E-02
**TACR1**	Tachykinin receptor 1	-3.218	5.28E-12	-1.882	2.76E-04
**TLR3**	Toll like receptor 3	2.61	9.33E-19	2.84	4.30E-15
**TLR7**	Toll like receptor 7	2.36	2.04E-11	2.48	1.60E-08
**ITGA3**	Integrin subunit alpha 3	2.96	4.11E-157	2.32	2.98E-80
**MYD88**	MYD88 innate immune signal transduction adaptor	1.528	4.76E-03	ns	ns
**LBP**	Lipopolysaccharide binding protein	4.96	1.06E-02	5.841	1.23E-03

ns: no significant p-values

Supplementary Table 7: Commonly expressed genes across the top predicted canonical pathways in unexposed 3D SYNs.

Gene	Occurrences	Present In
IL6	4	S100, PICS, WH, HIF1α
JUN	4	S100, PICS, WH, HIF1α
PDGFC	4	S100, PICS, WH, HIF1α
CCR1	3	S100, PICS, PF
CXCL8	3	S100, PICS, WH
MAPK4	3	S100, PICS, PF
MAPK8	3	S100, PICS, WH
MMP1	3	S100, WH, HIF1α
MYD88	3	S100, PICS, PF
NOS2	3	S100, PICS, HIF1α
PRKCG	3	S100, WH, HIF1α
PRKCQ	3	S100, WH, HIF1α
PRKD1	3	S100, WH, HIF1α
RAC2	3	S100, WH, HIF1α
RASD1	3	PF, WH, HIF1α
RASD2	3	PF, WH, HIF1α
RRAS	3	PF, WH, HIF1α
ACKR3	2	S100, PF
ADGRA2	2	S100, PF
ADGRB1	2	S100, PF
ADGRB2	2	S100, PF
ADGRE2	2	S100, PF
ADGRE5	2	S100, PF
ADGRF4	2	S100, PF
ADGRF5	2	S100, PF
ADORA1	2	S100, PF
ADRA2B	2	S100, PF
ADRA2C	2	S100, PF
BDKRB2	2	S100, PF
C3	2	CS, PF
CAMK2B	2	S100, HIF1α
CASP3	2	S100, CS
CCKBR	2	S100, PF
CCL20	2	S100, CS
CD70	2	CS, WH
CDKN1A	2	S100, HIF1α
CLCF1	2	CS, WH
CLEC7A	2	CS, PF
COL15A1	2	CS, WH
COL16A1	2	CS, WH
COL17A1	2	CS, WH
COL18A1	2	CS, WH
COL1A2	2	CS, WH
COL21A1	2	CS, WH
COL2A1	2	CS, WH
COL3A1	2	CS, WH
COL5A1	2	CS, WH
COL6A1	2	CS, WH
COL6A2	2	CS, WH
COL6A3	2	CS, WH
COL7A1	2	CS, WH
CXCR6	2	S100, PF
EGFR	2	S100, WH
FCGR2B	2	S100, PF
FGF2	2	WH, HIF1α
FGFR2	2	S100, WH
FZD10	2	S100, PF

Gene	Occurrences	Present In
GALR2	2	S100, PF
GIPR	2	S100, PF
GPR137C	2	S100, PF
GPR146	2	S100, PF
GPR155	2	S100, PF
GPR157	2	S100, PF
GPR161	2	S100, PF
GPR173	2	S100, PF
GPR176	2	S100, PF
GPR32	2	S100, PF
GPR37L1	2	S100, PF
GPR63	2	S100, PF
GPR87	2	S100, PF
GPRC5A	2	S100, PF
GPRC5B	2	S100, PF
GPRC5C	2	S100, PF
HCAR1	2	S100, PF
HCAR2	2	S100, PF
HRH1	2	S100, PF
IL15	2	CS, WH
IL17C	2	CS, WH
IL1RAP	2	CS, WH
IL6R	2	CS, HIF1α
ITGA3	2	PF, WH
KISS1R	2	S100, PF
LEP	2	CS, WH
LIF	2	CS, WH
LPAR1	2	S100, PF
LPAR3	2	S100, PF
LPAR5	2	S100, PF
LPAR6	2	S100, PF
MAPK11	2	S100, CS
MAPK13	2	S100, CS
MC1R	2	S100, PF
MMP11	2	S100, HIF1α
MMP12	2	S100, HIF1α
MMP19	2	S100, HIF1α
MMP24	2	S100, HIF1α
MMP25	2	S100, HIF1α
MTNR1B	2	S100, PF
NGFR	2	CS, WH
P2RY2	2	S100, PF
PLA2G4A	2	S100, PF
PLA2G4B	2	S100, PF
PLA2G4D	2	S100, PF
PLA2G4E	2	S100, PF
PLA2G4F	2	S100, PF
PTAFR	2	S100, PF
TACR1	2	S100, PF
TLR3	2	CS, PF
TLR7	2	CS, PF
TNFRSF1A	2	CS, WH
TNFRSF1B	2	CS, WH
TNFSF10	2	CS, WH
TNFSF13	2	CS, WH
TNFSF14	2	CS, WH
TNFSF9	2	CS, WH
VIM	2	WH, HIF1α

S100: S100 Protein Family; CS: Pathogen Induced Cytokine Storm;
HIF1a: HIF1a Signaling; PF: Phagosome Formation; WH: Wound Healing

Supplementary Table 8: Commonly expressed genes across the top predicted canonical pathways in *T. cruzi*-exposed 3D SYNs.

Gene	Occurrences	Present In
CXCL8	3	S100, CS, WH
EGF	3	S100, WH, HIF1α
HRAS	3	PF, WH, HIF1α
MAPK4	3	S100, CS, PF
MAPK8	3	S100, CS, WH
NOS2	3	S100, CS, HIF1α
PLCG2	3	S100, PF, HIF1α
PRKCG	3	S100, PF, HIF1α
PRKCZ	3	S100, PF, HIF1α
PRKD1	3	S100, PF, HIF1α
RAC2	3	S100, PF, HIF1α
RASD1	3	PF, WH, HIF1α
RASD2	3	PF, WH, HIF1α
RRAS	3	PF, WH, HIF1α
STAT3	3	S100, CS, HIF1α
ACKR3	2	S100, PF
ADGRA2	2	S100, PF
ADGRB1	2	S100, PF
ADGRB2	2	S100, PF
ADGRE2	2	S100, PF
ADGRE5	2	S100, PF
ADGRF4	2	S100, PF
ADGRF5	2	S100, PF
ADGRG3	2	S100, PF
ADGRG7	2	S100, PF
ADGRL1	2	S100, PF
ADGRV1	2	S100, PF
ADRA2B	2	S100, PF
ADRA2C	2	S100, PF
ADRB2	2	S100, PF
AVPR2	2	S100, PF
BDKRB2	2	S100, PF
CAMK2B	2	S100, HIF1α
CASP3	2	S100, CS
CCKBR	2	S100, PF
CCL20	2	S100, CS
CD70	2	CS, WH
CHRM4	2	S100, PF
CLCF1	2	CS, WH
CLEC7A	2	CS, PF
COL12A1	2	CS, WH
COL16A1	2	CS, WH
COL17A1	2	CS, WH
COL18A1	2	CS, WH
COL21A1	2	CS, WH
COL24A1	2	CS, WH
COL2A1	2	CS, WH
COL5A1	2	CS, WH
COL6A1	2	CS, WH
COL6A2	2	CS, WH
COL6A3	2	CS, WH
COL7A1	2	CS, WH
COL8A2	2	CS, WH
CXCR6	2	S100, PF
DRD4	2	S100, PF
EDA	2	CS, WH
EGFR	2	S100, WH
FCGR2B	2	S100, PF
FGF2	2	WH, HIF1 α
FN1	2	PF, WH
FSHR	2	S100, PF

Gene	Occurrences	Present In
FZD10	2	S100, PF
FZD9	2	S100, PF
GPR137C	2	S100, PF
GPR146	2	S100, PF
GPR155	2	S100, PF
GPR157	2	S100, PF
GPR161	2	S100, PF
GPR173	2	S100, PF
GPR176	2	S100, PF
GPR32	2	S100, PF
GPR37L1	2	S100, PF
GPR68	2	S100, PF
GPR78	2	S100, PF
GPR87	2	S100, PF
GPRC5A	2	S100, PF
GPRC5B	2	S100, PF
GPRC5C	2	S100, PF
HCAR1	2	S100, PF
HRH1	2	S100, PF
IL11	2	CS, WH
IL15	2	CS, WH
IL17C	2	CS, WH
IL1R2	2	CS, WH
IL1RAP	2	CS, WH
IL23A	2	S100, CS
IL6R	2	CS, HIF1α
ITGA3	2	PF, WH
KISS1R	2	S100, PF
LEP	2	CS, WH
LIF	2	CS, WH
LPAR1	2	S100, PF
LPAR3	2	S100, PF
LPAR5	2	S100, PF
MAPK11	2	S100, CS
MAPK13	2	S100, CS
MC1R	2	S100, PF
MMP11	2	S100, HIF1α
MMP12	2	S100, HIF1α
MMP17	2	S100, HIF1α
MMP19	2	S100, HIF1α
MMP24	2	S100, HIF1α
MMP25	2	S100, HIF1α
MTNR1B	2	S100, PF
NGFR	2	CS, WH
NPFFR2	2	S100, PF
OPN1MW	2	S100, PF
P2RY2	2	S100, PF
PLA2G4A	2	S100, PF
PLA2G4C	2	S100, PF
PLA2G4D	2	S100, PF
PLA2G4F	2	S100, PF
PLA2G6	2	S100, PF
PTAFR	2	S100, PF
TACR1	2	S100, PF
TACR3	2	S100, PF
TBXA2R	2	S100, PF
TLR3	2	CS, PF
TNFRSF1B	2	CS, WH
TNFSF10	2	CS, WH
TNFSF9	2	CS, WH
VIM	2	WH, HIF1α
XCR1	2	S100, PF

S100: S100 Protein Family; CS: Pathogen Induced Cytokine Storm;
HIF1a: HIF1a Signaling; PF: Phagosome Formation; WH: Wound Healing

Supplementary Table 9: Differential Expression Analysis of FOSL1 and JUN

Symbol	Gene Name	Unexposed		*T. cruzi*-exposed	
		Fold Change (log_2)	p-value	Fold Change (log_2)	p-value
**FOSL1**	FOS Like 1	3.52	2.01×10^{-24}	3.60	6.67E-21
**JUN**	Jun Oncogene	4.15	1.06×10^{-62}	4.22	4.16E-81
