# Supplementary material for: The transcriptome landscape of 3D-cultured placental trophoblasts reveals activation of TLR2 and TLR3/7 in response to low Trypanosoma cruzi parasite exposure
Source: Front Microbiol. 2023 Sep 20;14:1256385. doi: 10.3389/fmicb.2023.1256385 (PMC10548471; doi:10.3389/fmicb.2023.1256385)
Supplement: Supplementary file 2 [file Data_Sheet_2.PDF]

Supplementary Figures

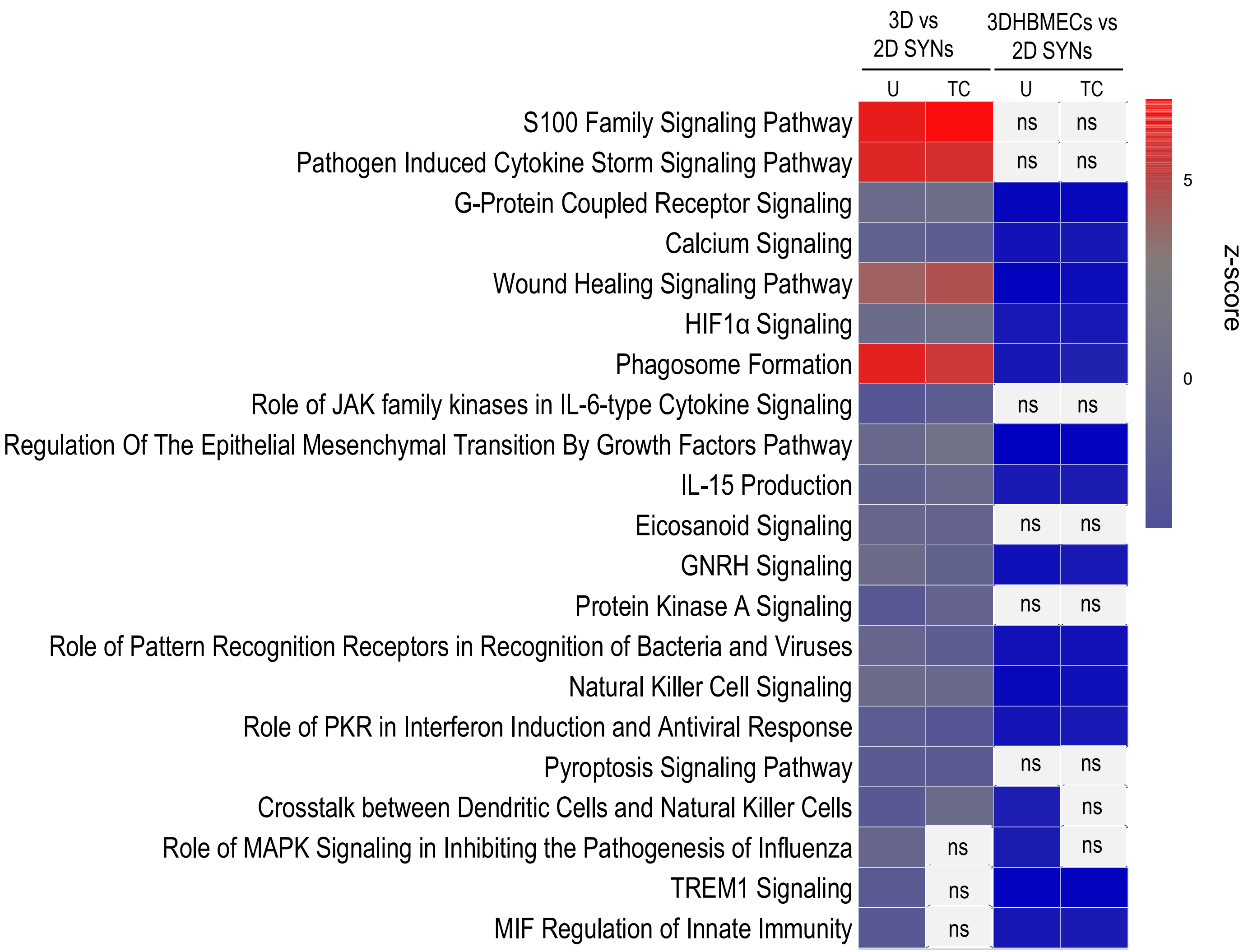

Supplementary Figure 1. Heat-map showing the activation z-scores of the top canonical pathways in unexposed and *T. cruzi*-exposed cultures, comparing 3D vs 2D SYNs, and 3D HBMEC vs 2D SYNs. Only pathways with z-score ≥ 2 and B-H p-value ≥ 0.05 were considered significant. ns: no significant B-H p-values.

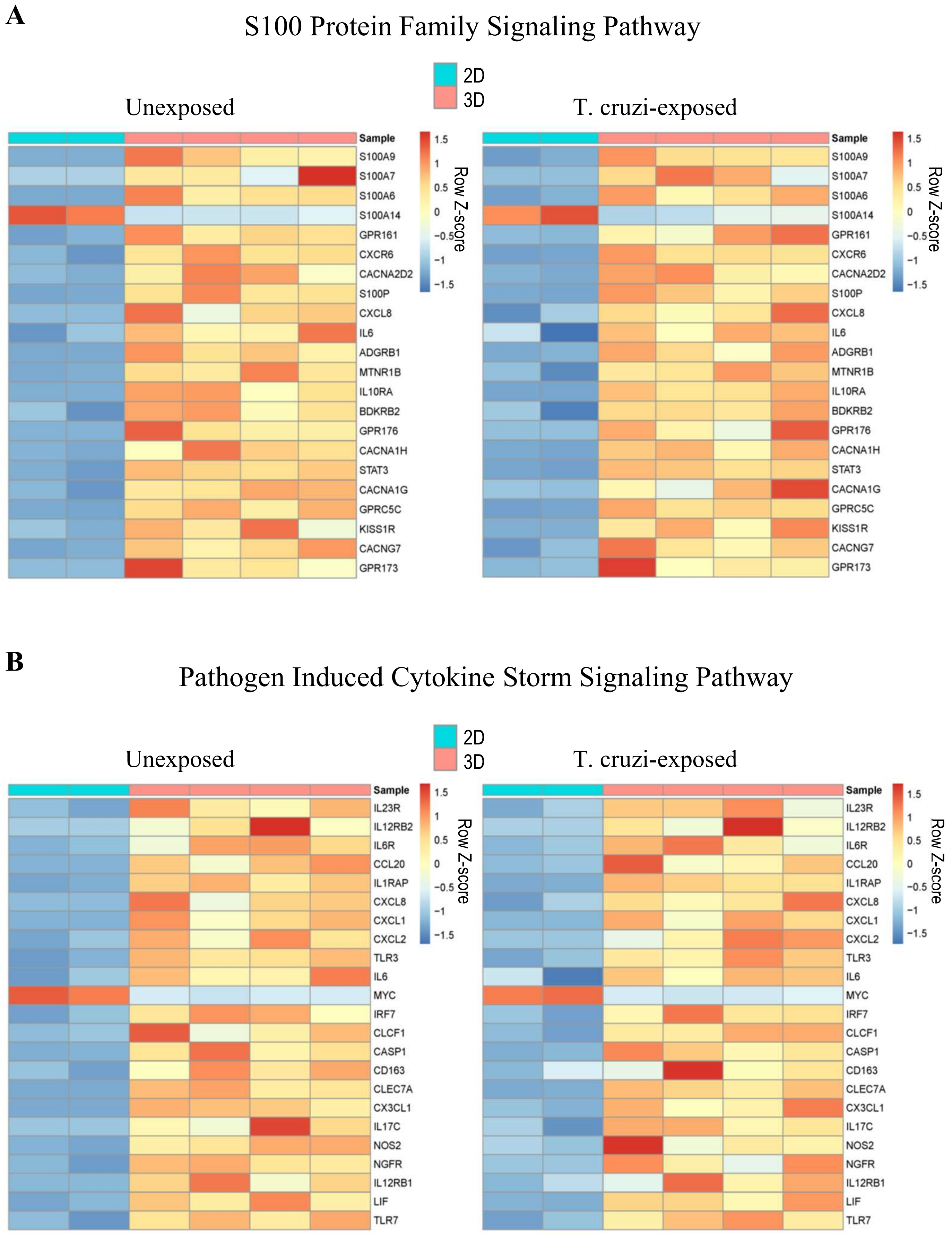

A
S100 Protein Family Signaling Pathway
2D
3D
Unexposed
T. cruzi-exposed
Sample
S100A9
S100A7
S100A6
S100A14
GPR161
CXCR6
CACNA2D2
S100P
CXCL8
IL6
ADGRB1
MTNR1B
IL10RA
BDKRB2
GPR176
CACNA1H
STAT3
CACNA1G
GPRC5C
KISS1R
CACNG7
GPR173
Row Z-score
1.5
1
0.5
0
−0.5
−1
−1.5
B
Pathogen Induced Cytokine Storm Signaling Pathway
2D
3D
Unexposed
T. cruzi-exposed
Sample
IL23R
IL12RB2
IL6R
CCL20
IL1RAP
CXCL8
CXCL1
CXCL2
TLR3
IL6
MYC
IRF7
CLCF1
CASP1
CD163
CLEC7A
CX3CL1
IL17C
NOS2
NGFR
IL12RB1
LIF
TLR7
Row Z-score
1.5
1
0.5
0
−0.5
−1
−1.5

C

Wound Healing Signaling Pathway

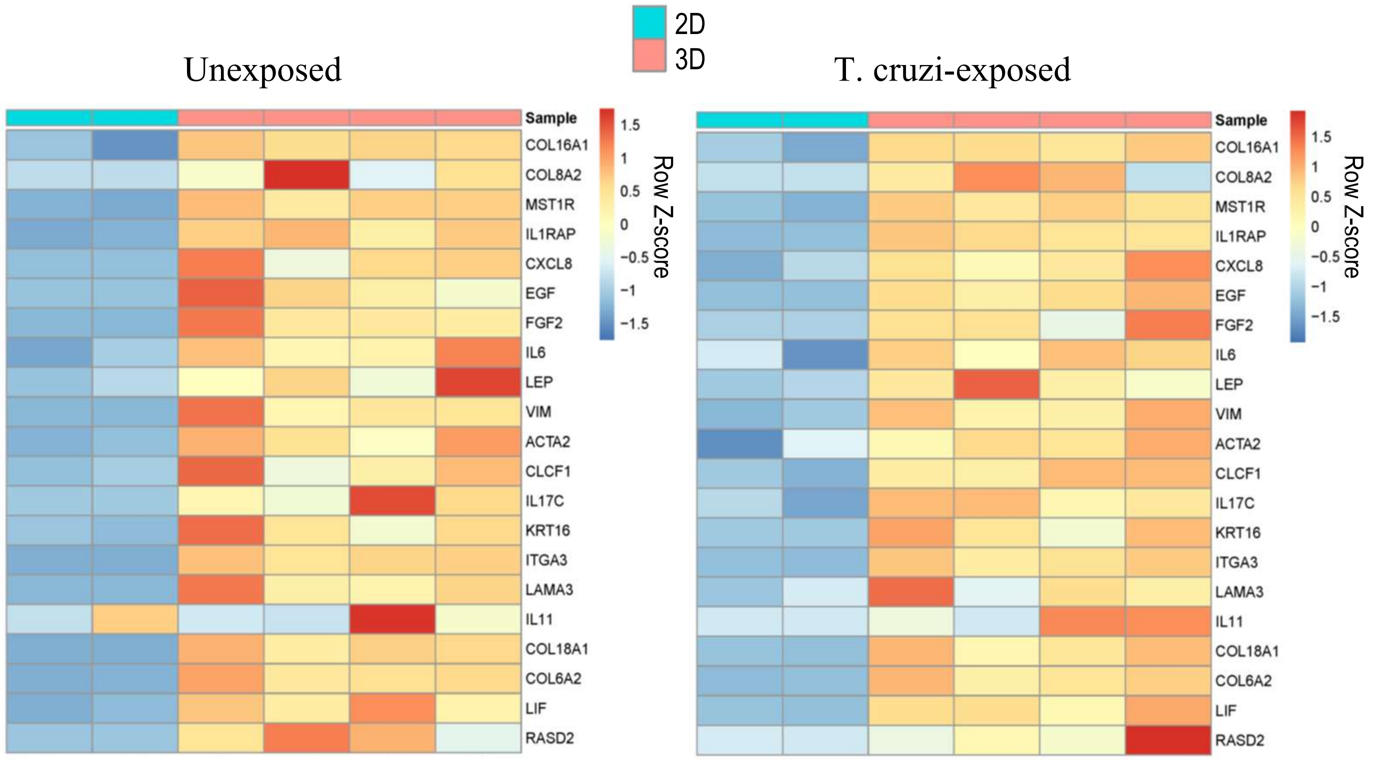

D

HIF1α Signaling Pathway

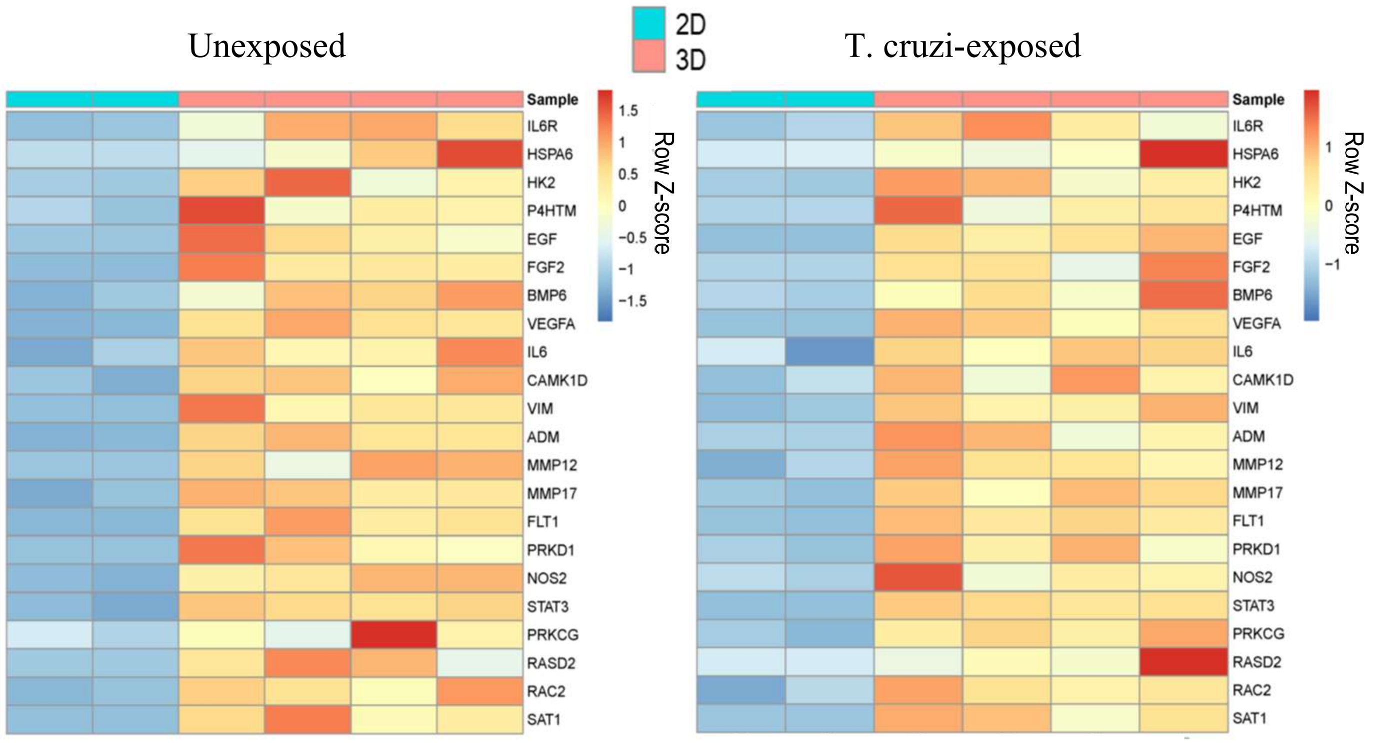

E Phagosome Formation Signaling Pathway

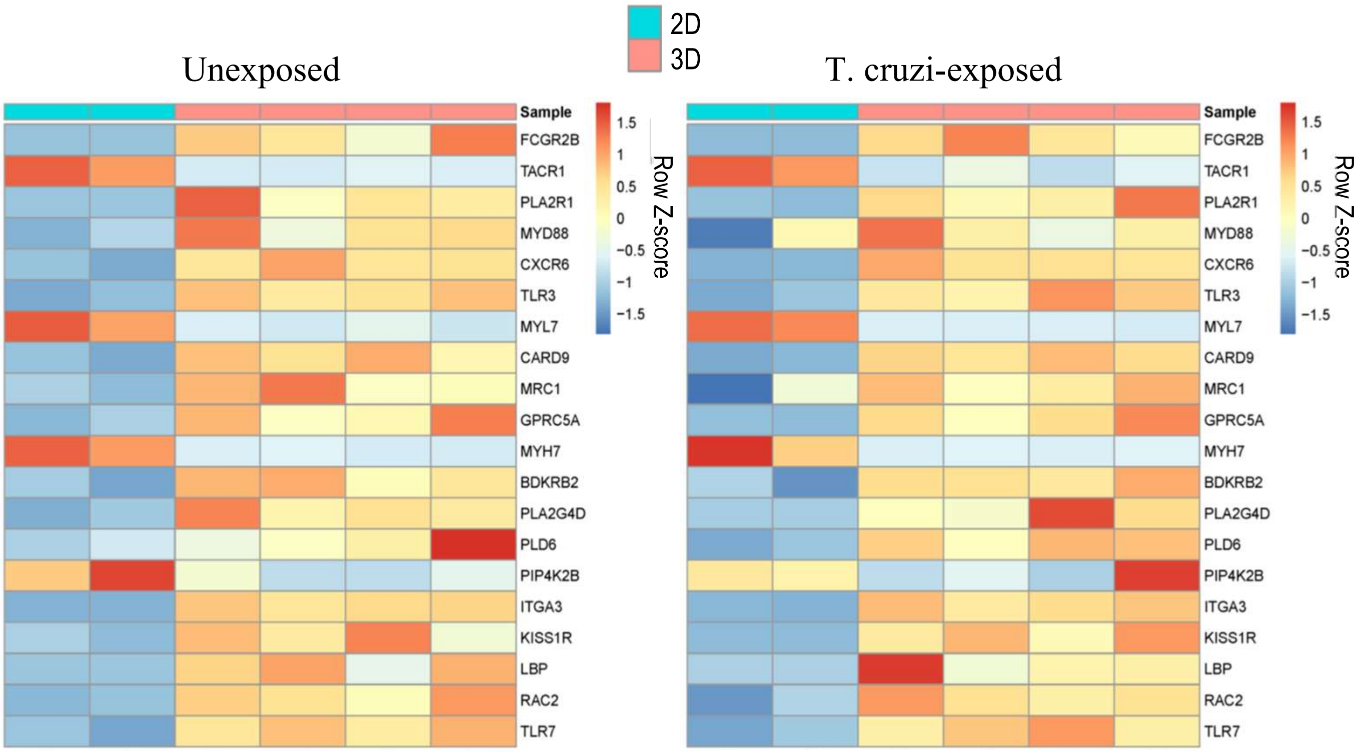

Supplementary Figure 2. Heatmap of normalized read counts of differentially expressed genes between 3D and 2D SYNs unexposed or exposed to *T. cruzi*. The heatmaps show selected genes in the top five canonical pathways.

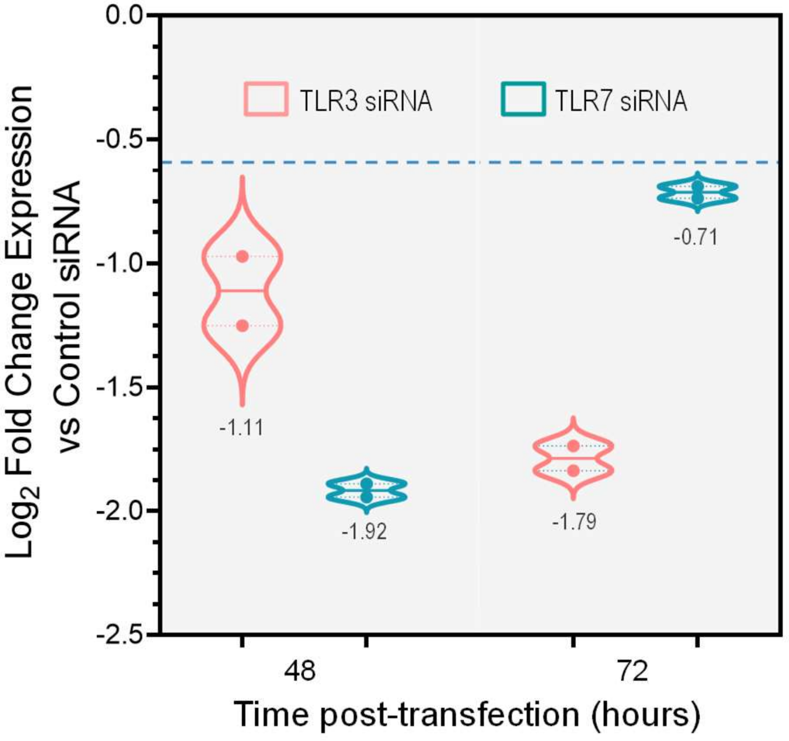

Supplementary Figure 3. Quantitative RT-PCR to verify TLR3 and TLR7 silencing. 2D SYNS were seeded into each well of 96-well plate and transfected with 50 nM of TLR3 siRNA and 50 nM of TLR7 siRNA mixed with 0.3 µl/well of DharmaFect 3 transfection reagent. Cells from triplicate wells were pooled and total RNA was extracted at 48 and 72 hs post-transfection. Violin-plot distributions show the $\log_2$ fold change in gene TLRs expression in 3D SYNs compared to 2D SYNs as determined by the ΔC_t method. Samples were normalized to GAPDH**.** Dotted lines represent first and third quartiles, and solid lines indicate the median (values shown below the plots). The dashed blue line indicates the assay cut off value (-0.58).
